# Syndromic cholera diagnosis masks diverse causes of diarrhoeal disease in Burundi revealed by portable metagenomics

**DOI:** 10.1371/journal.pntd.0014175

**Published:** 2026-09-01

**Authors:** Emilie Egholm Bruun Jensen, Néhémie Nzoyikorera, Mirena Ivanova, Pimlapas Leekitcharoenphon, Marie Noelle Uwineza, Idrissa Diawara, Joseph Nyandwi, Frank M. Aarestrup, Saria Otani

**Affiliations:** 1 Research Group for Genomic Epidemiology, National Food Institute, Technical University of Denmark, Kgs Lyngby, Denmark; 2 National Reference Laboratory, Institut National de Santé Publique (INSP) du Burundi, Bujumbura, Burundi; 3 Research Laboratory of Microbiology, Infectious Diseases, Allergology and Pathogen Surveillance (LARMIAS), Mohammed VI Faculty of Medicine, Mohammed VI University of Sciences and Health (UM6SS), Casablanca, Morocco; 4 Mohammed VI Higher Institute of Biosciences and Biotechnologies, Mohammed VI University of Sciences and Health (UM6SS), Casablanca, Morocco; 5 Mohammed VI Center for Research and Innovation, Rabat, Morocco; 6 National Public Health Institute, Ministry of Public Health Bujumbura, Bujumbura, Burundi; 7 Faculté de Médecine, Université du Burundi, Bujumbura, Burundi; Wayne State University School of Medicine, UNITED STATES OF AMERICA

## Abstract

**Background:**

Cholera outbreaks remain a major public-health challenge in sub-Saharan Africa, where diagnostic capacity is limited and clinical case definitions are non-specific and re ly heavily on syndromic diagnosis. Rapid identification of *Vibrio cholerae* is critical, yet cholera-suspected diarrhoea can have multiple infectious causes not captured by targeted diagnostics.

**Methods:**

We evaluated a mobile, culture-independent metagenomic sequencing workflow for on-site detection of gastrointestinal pathogens directly from faecal samples in Burundi. The offline workflow combined long-read Oxford Nanopore Technologies (ONT) sequencing with rapid, laptop-based taxonomic and antimicrobial resistance (AMR) screening and was deployed across a health centre, a district hospital, and a refugee transit camp. The frontline and real-time results were verified using both conventional culturing and in-depth bioinformatic analyses.

**Results:**

*V. cholerae* signals were only detected in a subset of suspected cholera cases, while many samples were dominated by alternative bacterial taxa, most frequently *Escherichia coli*. *V. cholerae* abundance correlated strongly with detection of the C holera T oxin P hage CTXφ, supporting differentiation between toxigenic signal and background exposure. AMR genes were detected across samples, providing early situational insight into resistance determinants among gastrointestinal bacteria.

**Conclusions:**

Mobile, offline metagenomic sequencing enables rapid frontline characterization of gastrointestinal disease, especially cholera-suspected, in resource-limited settings and complements existing diagnostics by improving etiological resolution and outbreak response.

## Author summary

Cholera remains a major cause of severe diarrhoeal disease in many low-resource settings, where diagnosis often relies on symptoms and limited laboratory testing. However, patients with suspected cholera may instead harbour other enteric pathogens that are not detected by cholera-specific diagnostic tests. In this study, we evaluated a portable, sequencing-based approach that allows direct identification of pathogens from stool samples at the point of care, without the need for laboratory infrastructure, internet access, or culture. Using this approach in multiple settings in Burundi, including a health centre, hospital, and refugee camp, we found a subset of suspected cholera cases were associated with *Vibrio cholerae*. Other cases were also dominated by other bacteria, particularly diarrheagenic *Escherichia coli*. We also detected antimicrobial resistance genes across samples, providing additional information relevant for treatment and surveillance. Our findings demonstrate that mobile metagenomic sequencing can improve the identification of disease causes directly in outbreak settings and help distinguish true cholera cases from other gastrointestinal infections. This approach has the potential to strengthen outbreak response, improve patient management, and support more accurate disease surveillance in resource-limited environments.

## Introduction

Acute gastrointestinal infections, particularly cholera, remain a recurrent and rapidly escalating cause of watery diarrhoea in many parts of sub-Saharan Africa [1–3]. Outbreaks often emerge in settings with fragile water and sanitation infrastructure and can be amplified by displacement and constrained healthcare resources.

Because severe dehydration can develop within hours and cholera can spread rapidly in settings with inadequate water, sanitation and hygiene [4,5], outbreak control and patient management depend on timely identification of *Vibrio cholerae* and differentiation from other gastrointestinal illnesses with overlapping symptoms. At the same time, confirmation frequently relies on centralised laboratory capacity and sample transport from remote places, and under-testing can delay targeted public-health action.

Despite its clinical and public-health importance, rapid and accurate identification of *Vibrio cholerae* remains challenging in many endemic settings [1–3]. Standard and conventional culture requires selective media, time for incubation and large equipped laboratories that are often unavailable in peripheral health centres. Rapid diagnostic tests, while easier to deploy, are typically limited to detecting a narrow range of *V. cholerae* serogroups and do not capture other gastrointestinal pathogens that can cause clinically similar symptoms [6–8]. Symptom-based diagnoses cannot reliably distinguish cholera from other causes of acute diarrhoeal disease, [9] leading to under- or over-estimation of cases during outbreaks. In addition, this will not identify healthy carriers. Co-infections can also complicate clinical outcomes with pathogens like *Campylobacter*, Norovirus GI/GII, and Enterotoxigenic *Escherichia coli* (ETEC) shown to be prevalent in dual infections [10–12]. These limitations compromise case management, delay outbreak confirmation needed for effective public-health response.

To address these diagnostic constraints, there is growing interest in approaches that can provide rapid, culture-independent identification of gastrointestinal infection agents, [1–5,13] including *V. cholerae*, directly from clinical samples, while remaining deployable in resource-limited and outbreak-prone settings. An optimal frontline diagnostic workflow would operate without reliance on centralized laboratory infrastructure, cold-chain stability, continuous grid power, or internet connectivity, while still enabling broad detection of bacterial pathogens and preferably the associated antimicrobial resistance determinants. Recent advances in portable sequencing platforms have created new opportunities to move genomic diagnostics closer to the point of care, allowing real-time generation and analysis of pathogen data directly at outbreak sites and from the clinical samples without culturing [13–17]. A portable and cost-effective sequencing method has been introduced with the MinION from Oxford Nanopore Technologies (ONT) [18]. Over the years, the technology has improved significantly with the most recent MinION flow cell (R10.4.1) and the 14 chemistry library preparation have an increased accuracy of >99% [19]. Together with the real-time basecalling, the portable laptop-compatible sequencing allows for a mobile laboratory setup, well-equipped for detection of infectious diseases directly at the outbreak site. With a metagenomic approach, where no culturing is needed, long-read sequencing can be used for frontline, on-site pathogen and antimicrobial resistance (AMR) detection [18,20]. This can assist the healthcare professionals in treatment decisions and support timely public health interventions.

In Burundi, cholera (*Vibrio cholerae*) is considered an endemic disease, [21] but with occasional outbreaks associated with seasonality (the wet season creates conditions that promote the growth and survival of *V. cholerae*), inadequate sanitation, limited access to clean drinking water, and natural disasters [1]. Epidemic cholera is caused by two toxigenic serogroups O1 and O139, [22] but O139 has so far not been isolated in Burundi [21]. Similar to many countries in sub-Saharan Africa, Burundi faces several socioeconomic challenges that affect access to essential services, including healthcare, especially in rural and peri-urban areas [23]. A large proportion of the population lives in rural areas with limited infrastructure, and ongoing humanitarian pressure from internal displacement and refugee movements (app. 89.000 refugees and asylum seekers so far) [24] has further stretched already limited health resources [25]. Access to specialised laboratory diagnostics is largely centralized, with comprehensive testing services typically only available in Bujumbura, making timely confirmation of infections difficult for peripheral clinics. Travel times from rural and camp settings to reference laboratories can be long and unpredictable creating barriers to both routine and outbreak-responsive diagnostics.

The main objective of this field feasibility and technology-evaluation study was to determine whether a fully, offline laptop-based ONT metagenomic workflow can be executed to a pathogen and AMR call at the point of care without grid power or internet connection, and whether its frontline calls are concordant with conventional culture as well as comprehensive High Performance Computing (HPC)-based analysis of the same reads. While portable sequencing has increasingly been applied in outbreak investigations, bioinformatic analyses are typically performed off-site due to the computational demands of large bacterial reference databases. In this study, we demonstrate that reducing bacterial reference database complexity while retaining species-level coverage enables rapid, fully offline pathogen detection directly in the field. The secondary objectives included characterising alternative bacterial taxa in patients grouped as suspected cholera, to assess whether Cholera Toxin Phage (CTXφ) co-detection supports discrimination of toxigenic signal, and to describe associated AMR gene content.

## Materials and methods

### Ethics approval

The ethical approval to conduct this study was given by the National Ethics Committee in Burundi (Reference number: CNE/10/2024). The approval included all sample collection for the GREAT-LIFE project (Global Health European and Developing Countries Clinical Trials Partnership 3 (Global Health EDCTP3) Joint Undertaking programme), which include acquiring biological materials and their genetic content for the purpose of GREAT-LIFE project only, which this study is under. The national ethics committee and the ministry of health in Burundi provided a waiver for the consent from participants in this study after being anonymised. The collection of the faecal samples is non-invasive and were collected by health professionals at all sites. Samples were assigned unique study codes before transferring them to the research team of this study, which received only the coded samples and minimal clinical metadata, without names or other direct personal identifiers. The same approved procedures applied at all study sites, including the refugee transit camp. Environmental sampling was included within the approved study activities and did not require separate ethical approval.

### Study design, participant recruitment and sample collection

This study was conducted as a prospective, multi-site, field-feasibility and technology-evaluation study with single timepoint sampling during predefined field deployments. Participants were recruited using time-bounded, site-based availability sampling from eligible individuals present during each site visit and who were able to provide a faecal sample. The sample number therefore reflects the eligible participants and samples available during each field window, together with the operational capacity of the mobile workflow including cold chain availability and maintenance. Samples were not selected according to disease severity, pathogen status or subsequent sequencing results.

**Fig 1:**
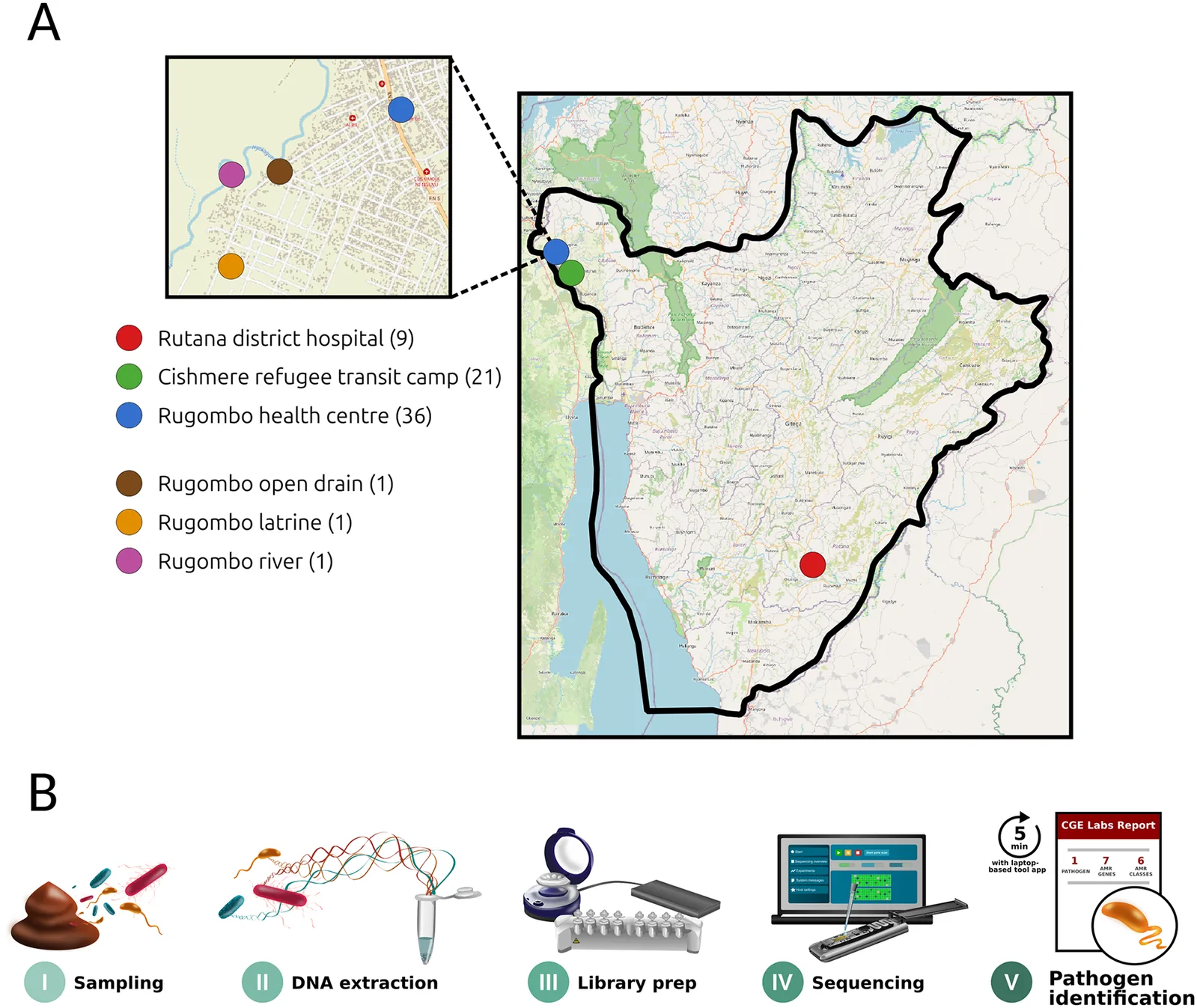
Study overview. **A)** Map showing the location of the metagenomic sample sites in Burundi. **B)** On-site rapid diagnostic workflow. This includes sample collection, extraction of DNA, library preparation, sequencing and basecalling, as well as pathogen and AMR identification using a local laptop-based solution. All conducted without access to grid power or internet. The map displaying the sites was created with the QGIS software [26] with basemap data from OpenStreetMap contributors available under the Open Database License and country boundary data from Natural Earth (https://www.naturalearthdata.com/downloads/) [27], which is also part of the public domain.

In September 2025, field visits were conducted at Rugombo Health Centre, which hosts the Cholera Treatment Centre (Fig 1A) to assess direct detection of *V. cholerae* in patient faecal samples. Patients suspected of *V. cholerae* infection were classified by the attending healthcare professional and managed through the Cholera Treatment Centre during the field-deployment period in Rugombo. The clinical routine classification was made independently of our arrival and before sequencing results were available. Stool appearance, dehydration status, symptom duration, treatment and prior antibiotic exposure was not recorded systematically due to the centre record system.

On-site metagenomic-based diagnostics was performed following our workflow (Fig 1B), including faecal sample collection, DNA extraction, library preparation, sequencing, (Graphics Processing Unit) GPU-enabled basecalling and rapid identification of pathogen and AMR locally using a laptop based solution. Faecal samples from asymptomatic companions (from the same household as the patients) were collected and analysed in parallel. Additionally, to explore potential environmental sources and exposure routes in the Rugombo area, environmental samples from the Mparambo neighbourhood in Rugombo were collected from accessible sites. This included samples from a communal latrine, from an open drain, and river water which served both for bathing and for water collection for household purposes.

The workflow was subsequently evaluated at two additional sites: Rutana District Hospital, and Cishemere refugee camp in Rutana Province (Fig 1A) to confirm the workflow efficacy and its feasibility across distinct clinical settings and pathogens.

At Cishemere refugee transit camp in Cibitoke (Fig 1A), faecal samples were collected from individuals self-reporting stomach issues, which included abdominal pain or discomfort at the time of sampling (no clinical diagnosis). Additionally, samples from individuals without reported stomach discomfort, abdominal pain, diarrhoea, vomiting or other gastrointestinal symptoms were collected (self-reported). At Rutana District Hospital, faecal samples were collected from admitted patients. The patients were classified by the attending clinical staff as presenting with a gastrointestinal complaint or illness. These samples were not intended to represent a single pathogen or illness, similar to the patients with cases of suspected cholera at Rugombo Heath Centre. S1 Table provides an overview of all samples analysed including the minimal metadata collected.

### ONT metagenomic sequencing

High molecular weight (HMW) DNA was extracted on-site from the faecal samples using Quick-DNA HMW Magbead Kit (Cat. No. D6060, Zymo Research) following the manufacturer’s instructions. The heatblock and centrifuge required for DNA extraction were powered using an external battery. The long-read library was prepared using the Ligation sequencing gDNA Native Barcoding Kit 24 V14 (SQK-NBD114.24, Oxford Nanopore Technologies). Enzymes and reagents which needed to be stored cold were kept on gel packs in a thermal container on-site. Samples were multiplexed and loaded onto the FLO-MIN114 flowcell (R10.4.1 chemistry). Sequencing was performed for a duration ranging from 20 to 45 hours, and the reads were basecalled on a GPU-enabled lap top using the high-accuracy basecalling option (model dna_r10.4.1_e8.2_400bps_hac@v5.0.0) from Dorado version 0.9.6 + 49e25e9. Demultiplexing and default quality control (Q-score >9, and minimum read length >200 bp) was carried out in MinKNOW version 25.05.14. Sequencing output statistics were calculated with NanoStat version 1.6.0. [28] and host reads were removed by Minimap2 version 2.28. [29] alignment against the Hg38 reference genome (GCF_000001405.40).

### Local laptop-based pathogen and AMR detection

For rapid and on-site taxonomic and AMR screening, demultiplexed FASTQ reads were concatenated and mapped with KMA (k-mer alignment) version 1.5.0. [30] KMA utilises ConClave scoring to resolve multiple template matches, which works well for redundant databases containing similar sequences [30]. A reduced-size bacterial reference database was used to enable the quick and offline analysis on the laptop. Database reduction was achieved by collapsing within-species strain diversity (i.e., selecting fewer representative genomes per species) while retaining species-level coverage. The reduced database contained 11,602 bacterial entries. AMR gene detection was performed using the entire AMR reference database ResFinder [31]. The alignment with KMA was performed with a minimum identity threshold of 25% (-ID 25) and a minimum depth threshold of 1 (-md 1) to retain candidate mappings for rapid on-site review, similar to Gand et al. [32] and others [33,34]. Here, the minimum template-identity parameter was kept loose and the resulting output was subsequently interpreted using combinations of template identity, depth and template length. This is useful for a frontline workflow, as this strategy avoids discarding potentially informative mappings at the initial computational stage, while allowing weak or ambiguous mappings to be classified as low-confidence results requiring further evaluation.

### Bacterial culturing and characterisation

To further confirm the presence of *V. cholerae* in the faecal samples from Rugombo, the samples were enriched in alkaline buffered peptone water (ABPW, pH 8.6 ± 0.2) prior to plating on thiosulphate citrate bile sucrose (TCBS) agar (Sigma) following the manufacturer’s instructions. After incubation, colonies displaying the expected colour (yellow) were selected as potential *V. cholerae* isolates. DNA was extracted and ONT libraries were prepared from those isolates as described before [35]. Assembly was performed with Flye version 2.9.6 (--nano-hq) and Medaka version 2.0.1. [36] was used to polish and create the consensus sequence (--model r1041_e82_400bps_hac_v5.0.0). One of the isolates (R24) had a sufficiently high amount of reads to close the genome. Trycycler version 0.5.4. [37] was used to circularise the genome. Assemblies from two isolates (R14, R23) were binned with MetaBat2 version 2.17. [38] to retrieve only pure and high completeness *V. cholerae* genomes. KmerFinder version 3.0.2 (database version: 2022-07-11) [30,39,40] was used to verify taxonomy, and sequence type (ST) and serotypes were identified with MLST version 2.0.9 (database version: 2025-09-15), [41] and CholeraeFinder version 1.0.[42]. The genomes were annotated with Bakta version 1.11.3 with database version 6.0. [43]. A phylogenetic tree based on core SNPs was created with CSI phylogeny version 1.4. [44] with *V. cholerae* N16961 as reference genome (accession no. NZ_CP028827.1).

### Comprehensive HPC-based analyses

To complement the local on-site laptop-based detection of pathogens and AMR genes, a more elaborate, and explorative command-line off-site approach was also applied using the HPC (high-performance computing) infrastructure at DTU. The concatenated FASTQ reads were mapped and aligned with KMA version 1.4.15 [30] to three reference databases: The first database was a full (not reduced) custom reference genomic database created from NCBI GenBank (downloaded and created 05.24.2022) and similar to Osakunor et al., [45] and Otani and Jespersen et al. [46]. It consisted of completed and draft bacterial genomes. The sequencing reads were also mapped to a custom viral database created from NCBI GenBank (downloaded 04.12.2025). A template coverage threshold of >50% was used for the alignments. Additionally, a *V. cholerae*-specific database was created from the five Burundi isolates from this study, as well as 48 genomes from the *V. cholerae* isolate collection from PubMLST (downloaded 12.02.2026) which passed the following criteria: source = human, number of contigs < 10, total length (Mbp) > 3.8, and N50 > 50.000. The reference genomes were padded with 800 characters of N’s to optimise KMA conclave scoring for resolving multiple template matches and better distinguish between isolates. The samples were screened for diarrheagenic *E. coli* virulence marker to confirm pathogenicity. First, the reads were mapped with KMA against the core Virulence Factor Database (VFDB, version from Jul 24 2026) [47]. Screening was performed for primary pathotype markers (*stx1*, *stx2*, *eae*, *bfpA*, *eltA*/*eltB*, *est*, *ipaH*, *aggR*) and supporting virulence markers (*ehxA*, *saa*, *aatA*, *aaiC*, *tir*, *escV*).

### Data analysis and software

Additional explorative analyses were performed. First, t he kbio.math.diversity.alpha python package [48] was used to describe alpha diversity by calculating richness (chao1), Simpson diversity index, and Shannon’ evenness. Beta-diversity was described with a compositional data analysis approach [49]. With python, the package pyCoDaMath [50] was used to perform zero replacement and transform the abundance tables to centered log-ratio (CLR)-values. The same package was used to create the PCA figures. Linear association between the abundance of bacteria and viruses was tested using Pearson’s correlation coefficient with the python SciPy library [51]. The basepair counts were normalised to adjust for reference length and bacterial depth using equation 2.


$$\begin{array}{c} B_{p_{norm}}=\frac{Total\;bp\;aligned\;to\;reference}{Reference\;length\;\cdot Total\;bacterial\;bp}\cdot {\text{1}\text{0}}^{\text{9}} \end{array}$$

**Equation 2.** Basepair normalisation to account for reference length and bacterial depth.

## Results

### Sequencing output

**Fig 2:**
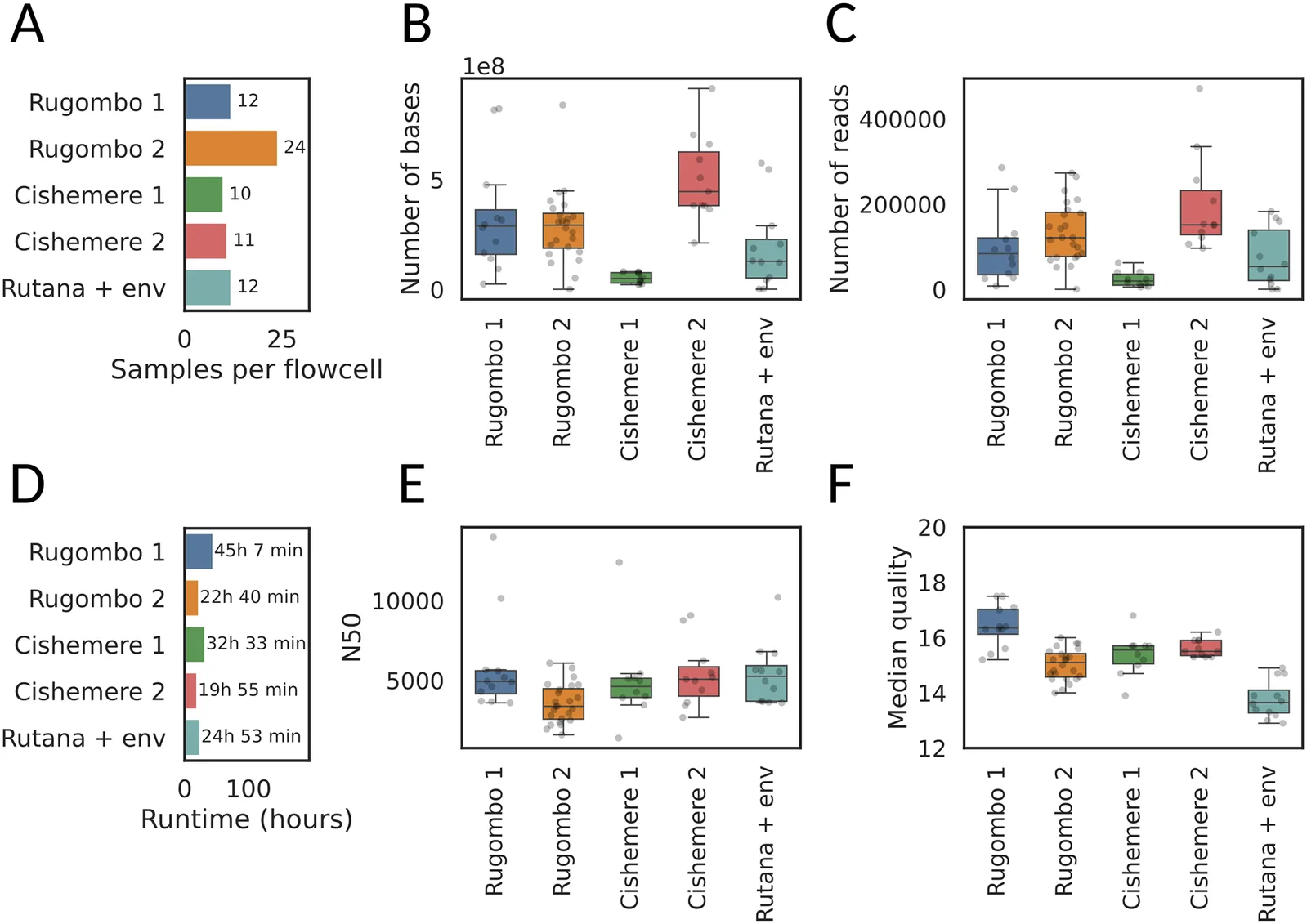
Sequencing output summary of the five flowcells with faecal samples. **A)** Number of samples barcoded per flowcell. **B)** Number of bases sequenced per sample per flowcell. **C)** Number of reads per sample per flowcell. **D)** Sequencing time. **E)** N50 per sample per flowcell **F)** Median quality per sample per flowcell.

Five FLO-MIN114 flowcells (R10.4.1 chemistry) were used for the metagenomic sequencing (Fig 2). The samples from Rugombo Health Centre were sequenced on two flowcells (based on the flowcell availability at that site at the moment), with 12 and 24 samples for 45 hours and 7 minutes, and 22 hours and 40 minutes, respectively. The flowcell with 12 samples yielded 3.97 giga bases (Gb), 1.2 M reads, a median quality of Q16.35, and a N50 of 4.93 kilo basepairs (bp). The second flowcell from Rugombo Health Centre with 24 samples yielded 6.83 Gb, 3.2 M reads, a median quality of Q15.10, and a N50 of 3.36 kb.

The 21 samples from Cishemere refugee camp were sequenced on two flowcells for 32 hours and 33 minutes, and 10 hours and 55 minutes respectively. The first flowcell yielded 0.52 Gb, 0.2 M reads, a median quality of Q15.55, and a N50 of 4.62 kb. The second flowcell yielded 5.55 Gb, 2.2 M reads, a median quality of Q15.5, and a N50 of 5.07 kb. The nine samples from Rutana District Hospital and the three environmental samples from the Mparambo neighbourhood in Rugombo were multiplexed and sequenced on one flowcell for 24 hours and 53 minutes, and yielding 2.28 Gb, 0.9 M reads, a median quality of Q13.65, and a N50 of 5.26 kb. Although the total sequencing runs lasted 11–45 hours, the major frontline pathogen and AMR profiles were available within approximately 12 hours. Sequencing was continued to increase read depth and improve the characterisation of lower-abundance signals. All flowcell outputs are summarised in S2 Table as well.

### Metagenomic mobile laboratory on-site diagnostics of *V. cholerae* at Rugombo Health Centre

**Fig 3:**
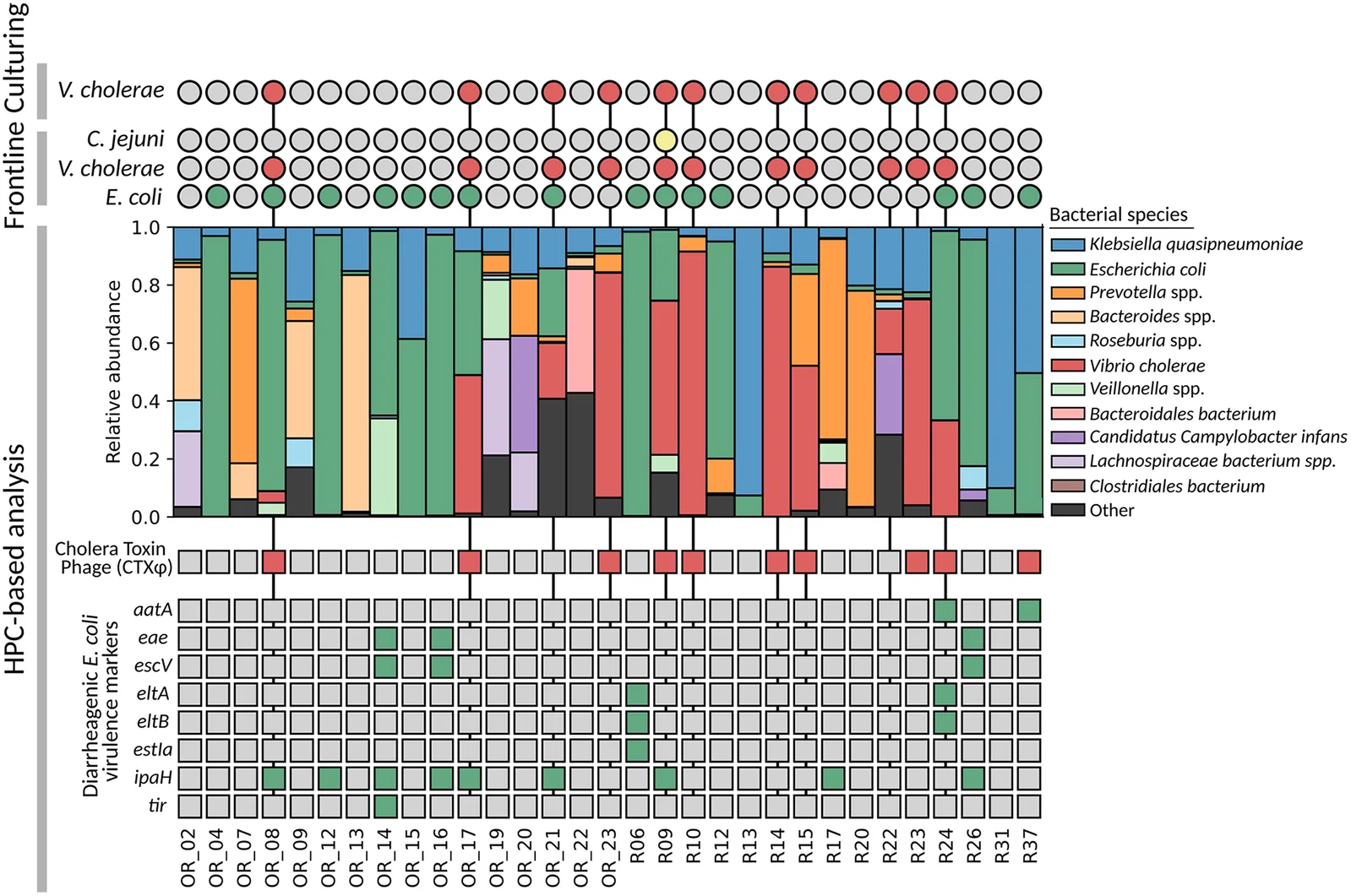
Metagenomic insight into the faecal samples collected from patients with *Vibrio cholerae*-suspected infections from Rugombo Health Centre. The figure shows a comparison between culturing on TCBS plate agar for *V. cholerae* detection, frontline laptop-based diagnostics results with a reduced bacterial database, and a HPC-based analysis utilising a comprehensive database. The first two panels show presence/absence of the gastrointestinal pathogen from the culturing and frontline laptop-based alignment respectively. Red indicates detection of *V. cholerae*, green indicates *E. coli*, and yellow indicates *C. jejuni*. Underneath, the barplot shows the in-depth compositional analysis of the gut microbiome obtained from the alignment to the full comprehensive database. The HPC-based analysis also includes Cholera Toxin Phage (CTXφ) screening (red indicates positive detection), as well as screening for diarrheagenic *E. coli* virulence markers (green indicates positive detection).

At the Rugombo Health Centre, 31 samples from patients with a suspected *V. cholerae* infection were collected, as well as five samples from patient household companions (with no gastrointestinal symptoms, and three environmental samples from accessible sites. More sites were not possible due to physical accessibility issues during the field visit. S1A Fig shows the workflow in an STARD-like diagram. *V. cholerae* was detected in high abundance in 11 of the 31 patient samples (Fig 3). In six of these *V. cholerae*-positive cases, high abundance of *E. coli* was also observed, and in one of the samples (R09) *V. cholerae*, *E. coli*, and *Campylobacter jejuni* were detected (Fig 3), with *C. jejuni* being low abundant (i.e., 0.976% of the sequenced bacterial basepairs) Nine samples had *E. coli* as the only potential pathogen detected (Fig 3, S3 Table). These lap top based results were subsequently confirmed by alignment to the full comprehensive genomic database (Fig 3). To determine whether the high-abundance *E. coli* was accompanied by evidence of diarrhoeagenic potential, metagenomic reads were screened for pathotype-associated virulence markers. Such virulence markers were detected in 11 of the 15 samples where *E. coli* was observed, supporting the presence of pathogenic *E. coli* strains (Fig 3, S3 Table). The ipaH marker, which is associated with *Shigella* spp. and Enteroinvasive *E. coli* (EIEC) and cannot be distinguished between Shigella and EIEC [69,70], was detected in eight samples, including three that also carried the Locus of Enterocyte Effacement (LEE)-associated markers (*eae*, *escV*, and *tir*) [71,72]. Enterotoxigenic *E. coli* (ETEC)-associated markers (*eltA*, *eltB*, and *estIa*) [73,74] were detected in two samples, and the Enteroaggregative *E. coli* EAEC-associated marker *aatA* in one. These findings support the presence of diarrhoeagenic *E. coli* signatures in a subset of samples, although they do not establish strain-level pathotypes or causation.

Two samples (OR_20 and R22) had high abundance of *Candidatus Campylobacter infans* (Fig 3), which has been shown to be present in both symptomatic and asymptomatic children under two years with diarrhoea, [52] and is therefore not regarded as a causative agent. Cholerae Toxin Phage toxin (CTXφ) were detected in all but one of samples positive of *V. cholerae*, further supporting *V. cholerae* as a causative pathogen (Fig 3, S3 Table). No apparent potential pathogens were detected in the remaining 10 patient samples (Fig 3). However, remaining commensal-associated African microbiome members [46] were detected in all the samples, e.g., *Bacteroides*, *Blautia*, *Roseburia* and *Prevotella* (Fig 3). Based on taxonomic composition (not excluding strain-specific or opportunistic pathogen potential, no candidate primary gastrointestinal pathogens were detected in any of the asymptomatic companions (S2A Fig, S3 Table). The gut microbiome of these individuals were characterised by a high abundance of *Klebsiella quasipneumoniae*, formerly named *Prevotella*-species like *Segatella hominis* (formerly *Prevotella hominis*) and *Leyella stercorea* (formerly *Prevotella stercorea*), and *Escherichia coli* (S4 Table)*.* Faecal indicator bacteria such as *Klebsiella quasipneumoniae*, *S. hominis*, and *E. coli* were also found in high abundance in the open drain and river environmental samples (S5 Table, S2B Fig). The latrine microbiome showed high similarity to the asymptomatic companions, whereas the river and open drain sample clustered within the patient samples (S2C Fig). A list of all unique bacterial species identified with the alignment to the comprehensive genomic database can be found in S6 Table.

AMR genes identified in patients included resistance to several antimicrobial classes, with the following being the most dominant: tetracyclines (e.g., *tet(Q)*, *tet(W)*, and *tet(O)*, beta-lactams (e.g. *cfxA*-variants, *bla*TEM, and *bla*CTX-M) and macrolides (e.g., *erm(F)*, *mph(A)*, and *mph(E)*) (S3B Fig, S3 Table). Additional alignment to a reference virus database was performed. However, none of the patient samples had reads mapping to common diarrhoeal-causing viruses such as adenoviruses (S5 Fig).

### Isolation of *V. cholerae* from rugombo health centre

Presence of *V. cholerae* was supported by bacterial culturing from the faecal sample (S3 and S7 Table), however as the sample size is small this explorative agreement analysis does not constitute a formal diagnostic-accuracy evaluation. The 11 faecal samples positive of *V. cholerae* from the on-site alignment also showed morphologically positive *V. cholerae* colonies on TCBS agar (yellow colonies). Those were OR_08, OR17, OR_21, OR_23, R9, R10, R14, R15, R22, R23 and R24. All of those presumptive TCBS *V. cholera*e-positive isolates except one (OR_17 - DNA was degraded) were also whole genome sequenced (WGS) with long reads. Five of which were identified as pure *V. cholerae* (Table 1), while the remaining five isolates were identified as a mixture of different taxa, yet *V. cholerae* was the most abundant in the culture. Four of the isolates were ST69 with O1 and El Tor variant, and clustered together with a difference of 0–20 SNPs (S6 Fig). The non O1/O139 strain (R24) was circularised, and as expected, it clustered separately from the four other isolates (S5 Fig).

**Table 1:** Characteristics of the five *V. cholerae* strains.

Sample	No. of contigs	Total length (bp)	GC content (%)	ST type	Serogroup
R15	6	4,232,353	0.476	ST69	O1/El Tor variant
R10	3	4,224,272	0.476	ST69	O1/El Tor variant
R14	2	4,085,442	0.475	ST69	O1/El Tor variant
R23	60	3,937,251	0.476	ST69	O1/El Tor variant
R24	2	4,170,543	0.476	ST366	Non-O1/non-O139

To identify which *V. cholerae* strains were present in the metagenomic samples, a database of *V. cholerae* genomes was created from downloaded reference genomes of *V. cholerae*, as well as the five pure isolates from this study (see methods). Sequence alignment to this *V. cholerae*-specific database confirmed *V. cholerae* presence in the 11 cholerae-positive samples previously identified by frontline metagenomics and TCBS culturing (S8 Table). For all 11 samples, O1/El Tor variant from Burundi 2025 was the dominant one, while R24 had a mix of the O1/El Tor variant and the non-O1/non-O139, with the latter being in agreement with our WGS result.

### Pipeline validation at additional sites

**Fig 4:**
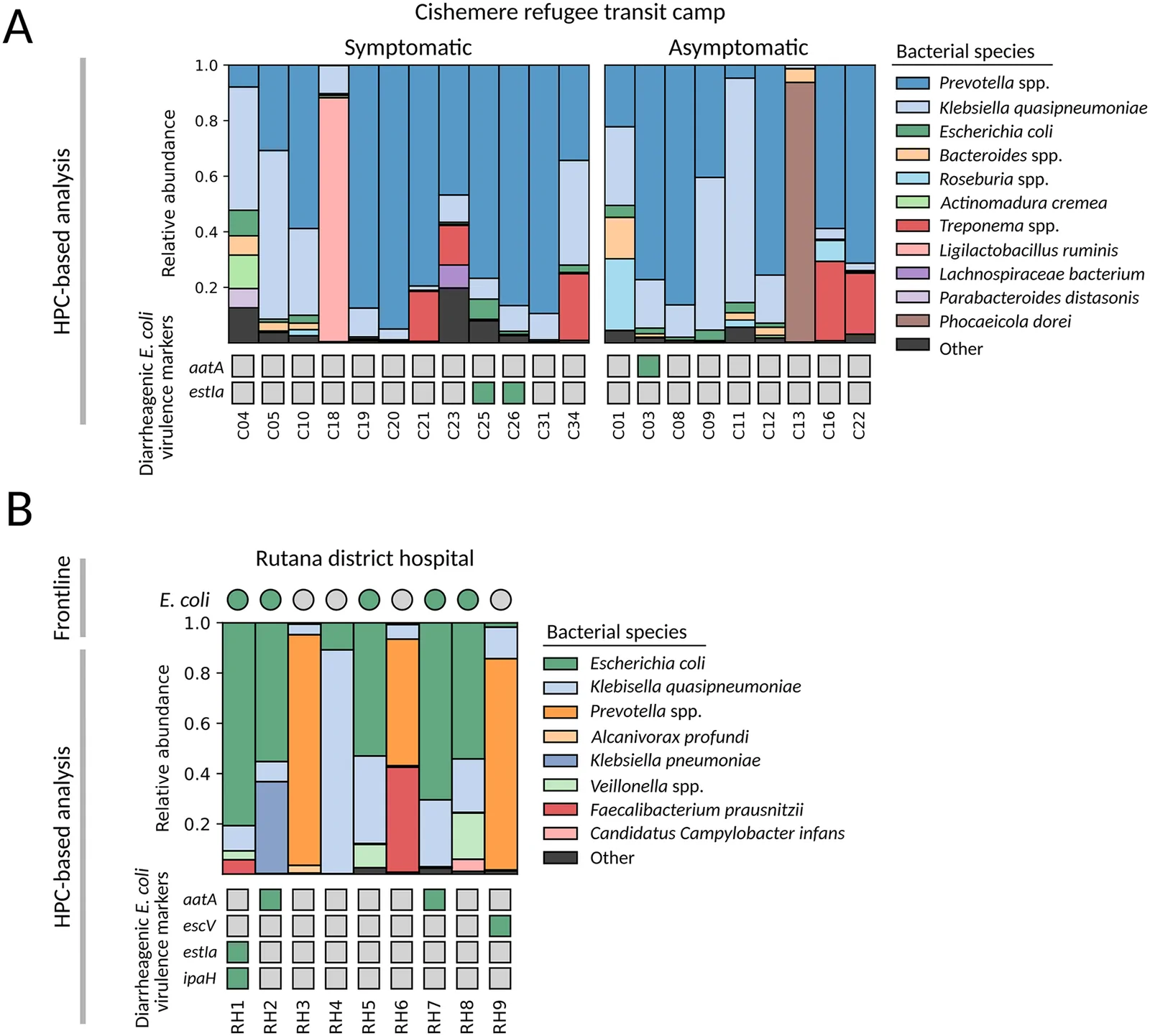
Metagenomic insight into the faecal samples collected from individuals with stomach discomfort and from asymptomatic individuals at the Cishemere refugee camp in Cibitoke, as well as from patients with suspected gastrointestinal infections at Rutana District Hospital. **A)** Bacterial species composition of the faecal samples collected from individuals at the Cishemere refugee transit camp. **B)** Comparison of the frontline diagnostic results at Rutana District Hospital obtained from the lap top based alignment against a reduced bacterial database with the in-depth command-line based approach with a comprehensive bacterial database. The result shows a presence/absence of the gastrointestinal pathogen from the on-site diagnostics, as well as a barplot showing the abundance of bacterial species within the sample from the command-line approach.

Two additional sites were also visited: Rutana District Hospital and Cishemere refugee transit camp in Cibitoke. S1 Fig shows the workflow in an STARD-like diagram. From Rutana District Hospital, nine faecal samples were collected from admitted patients with gastrointestinal symptoms. *E. coli* was identified as highly abundant in five of the nine samples, which was also found with the command-line alignment to the comprehensive genomic database (Fig 3C, S9 and S3 Tables). In three of the *E. coli*-positive samples, diarrheagenic virulence markers were detected (Fig 4). RH1 likely had a mixed ETEC (supported by *estIa*) and EIEC infection (supported by *ipaH*), and RH2 and RH7 both likely had an EAEC infection (supported by *aatA*) (S3 Table). In three of the four samples where *E. coli* was not the dominant species (RH3, RH6, and RH9), *Segatella copri* was found abundant. AMR genes identified in the Rutana samples included resistance to Beta-lactams (all samples, e.g. *cfxA*-variants, *bla*TEM, and *bla*CTX-M), as well as tetracyclines (e.g., *tet(Q), tet(B), and tet(W)*), aminoglycosides, macrolides, and quinolones (S3A Fig, S3 Table).

On-site at the Cishemere refugee camp in Cibitoke, 12 individuals who self-reported stomach pain or discomfort were collected. The faeces appeared compact and showed no signs indicating diarrhoea. Additional nine samples were also collected from nine self-reported asymptomatic individuals. No candidate primary gastrointestinal pathogens were detected using our on-site sequencing pipeline for any of the samples (Fig 4A). The in-depth analysis of the bacterial composition found no reads mapping to either *V. cholerae* or *C. jejuni*, and no difference between the individuals with stomach discomfort and asymptomatic individuals were identified either (S4 Fig). Screening of diarrheagenic virulence genes showed that symptomatic samples C25 and C26 were positive of *estIa*, however since only C25 had alignment to a *E. coli* chromosome (with low identity, coverage, depth, see S3 Table) in the comprehensive HPC alignment, no conclusion could be made.

The most abundant bacterial taxa in both symptomatic and asymptomatic individuals were *S. hominis*, *K. quasipneumoniae*, and *Leyella stercorea* (previously *Prevotella stercorea*) (S10 Table). Other dominant species were *Treponema* spp. and *Roseburia* spp. (Fig 4A), both reported common members of the sub-Saharan human gut microbiome [46]. Antimicrobial resistance patterns were identified within the samples: All individuals with stomach discomfort harboured genes encoding resistance to nitroimidazoles, beta-lactams, tetracyclines, and nitroimidazoles (S3C Fig). This resistance profile was also observed for most of the asymptomatic individuals. Additional alignment to a virus reference database (see methods) was performed to determine if the symptomatic individuals had a viral infection such as adenoviruses. However, no reads aligned to DNA viruses (S4C Fig).

### Presence of CTXφ confirms pathogenicity of *V. cholerae*

Investigations into the abundance correlation between bacteriophages and their bacterial hosts spanning all samples across all sites, revealed that the abundance of *V. cholerae* was positively correlated with the abundance of the filamentous bacteriophage Cholera Toxin Phage (CTXφ) (S7 Fig) with a Pearson correlation coefficient of 0.851. The pathogenicity of *V. cholerae* often comes from the integration of the Cholera Toxin Phage (CTXφ) into the genome [53].

The *E. coli* phage phiSTEC1575-Stx2k as well as the *E. coli* phage RCS47 were both found linearly associated with the abundance of *E. coli*, with a Pearson correlation coefficient of 0.850 and 0.498, respectively. The Stx2k phage carries Shiga toxin type 2 and is able to infect and integrate the gene into the genome, which convert non-pathogenic *E. coli* into a highly pathogenic and Shiga toxin-producing strain (STEC) [54]. The *E. coli*-infecting RCS47 P1-like bacteriophage have been reported in clinical *E. coli* strains and are associated with the beta-lactam resistance gene *bla*SHV-2 [55].

## Discussion

Here, we evaluated a fully mobile, offline ONT metagenomic workflow for rapid on-site detection of *V. cholerae* and other gastrointestinal pathogens directly from faecal samples in Burundi. The workflow was designed for outbreak-prone, resource-limited settings without access to grid power, internet or centralised laboratory infrastructure, and combines rapid, laptop-based screening with subsequent in-depth read mapping for confirmation. Across three distinct settings: A health centre evaluating suspected cholera cases, a district hospital, and a refugee transit camp, we demonstrate that culture-independent long-read metagenomics can generate actionable pathogen profiles on-site, in approximately 12 hours from sample to report, and reveal substantial heterogeneity in the aetiology of “cholera-suspected” gastrointestinal disease.

Conventional diagnostic methods, including rapid antigen tests targeting *V. cholerae* O1/O139, remain valuable tools for frontline *V. cholerae* detection. However, when rapid tests are negative, inconclusive, or when patients do not respond to treatment, untargeted metagenomic sequencing provides an important complementary approach by enabling broader detection of gastrointestinal pathogens without prior assumptions. In this context, ONT-based metagenomics can function as an escalation tool rather than a replacement for existing diagnostics, especially here where at least one of the *V. cholerae* cases was not O1/O139.

Only a subset of clinically suspected cholera cases showed high-abundance *V. cholerae* signals consistent with a causative role in such cases of diarrhoea, [56,57] while a large fraction of samples were dominated by other taxa, most frequently diarrheagenic *E. coli* (EIEC, EPEC/STEC, EAEC, ETEC). This is important for outbreak settings where symptom-based diagnosis is unavoidable but non-specific: Acute watery diarrhoea is clinically overlapping across multiple pathogens, and reliance on syndromic case definitions alone can both inflate cholera case counts and miss alternative causes requiring different clinical and public-health attention [6–8]. By providing an empirical profile from a single assay, metagenomics can support a “syndromic” diagnostic approach that is particularly relevant when conventional testing is unavailable or focused on a single target.

Detection does not necessarily imply causation, a challenge for metagenomic frontline diagnostics. In this study, applying a template coverage threshold of >50% removed low-abundance *V. cholerae* mappings and restricted detection to samples where the bacterium dominated the microbial signal and aligned with the clinical picture of suspected cholera. This highlights the importance of implementing explicit interpretation criteria when using metagenomics for pathogen detection directly from clinical samples. In clinical metagenomics, low-level sequence detection can arise from colonisation, transient exposure, or environmental contamination rather than representing the primary aetiology of disease [58]. For frontline detection pipelines, these findings support the use of abundance and coverage thresholds, contextual co-detections, and clinical plausibility rather than binary positive/negative reporting.

Using long-read metagenomics on-site captures not only taxonomic signals, but also pathogenicity-derived markers that can further support our interpretation. In our dataset, the abundance of *V. cholerae* correlated strongly with CTXφ abundance, providing internal biological support that high-abundance detections reflected toxigenic cholera-associated signals rather than low-level background. The *ctx* genes are carried by CTXφ and are central to epidemic cholera, [53] and incorporation of virulence gene evidence alongside species detection is a logical next step for metagenomic *V. cholerae* confirmation directly from stool. Similarly, pathogenic *E. coli* can be distinguished from commensal *E. coli* by incorporating detection of diarrheagenic virulence marker genes in the reads. More broadly, this illustrates how metagenomics can move beyond identifying taxa to supporting interpretation using biological context.

We also performed culture-based confirmation on a subset of samples and observed that selective enrichment and plating yielded presumptive colonies, but isolate purity remained variable, with WGS revealing mixed signals even from isolated colonies. This likely reflects the complexity of faecal material and the selectivity of differential media in field settings. This also supports a pragmatic view of culture confirmation during outbreak response: While culturing remains essential for phenotypic testing and downstream characterization, it can be slow and operationally constrained on-site, and it does not always provide immediate clarity at the point of care. Culture-independent sequencing can therefore complement culture by accelerating confirmation and guiding which samples should be prioritized for isolation, storage, and phenotypic work.

Metagenomic profiling revealed that cholera-positive patients exhibited reduced bacterial diversity compared to asymptomatic companions, consistent with previous observations that *V. cholerae* infection is associated with a collapsed gut microbiome [57,59]. Several commensal taxa were abundant in asymptomatic companions, including *Segatella hominis* and *Prevotella pectinova*. Previous studies have shown other commensal bacteria such as *Blautia* species can contribute to colonisation resistance against *V. cholerae* through interference with quorum sensing and degradation of host-derived virulence-inducing compounds [57,60]. However, no such evidence exists in the case of *S. hominis* or *P. pectinova*. Short-chain fatty acid-producing bacteria do however provide colonization resistance against *V. cholerae*, [61–63] and it can be speculated that the SCFAs produced by *S. hominis* [64,65] and *P. pectinovora* [66] provide similar protection. However further investigations are needed to confirm this.

Environmental sampling did not yield clear *V. cholerae* detections in our limited set of samples. However, the environmental metagenomes contained high abundances of faecal-associated taxa and potential indicator species, consistent with exposure to human waste. Environmental *V. cholerae* detection is inherently challenging because shedding is intermittent, pathogen abundance may be low relative to complex background communities, and sampling is sensitive to timing and hydrological conditions [67]. The absence of detections may be difficult to interpret, particularly given the small number of samples and field constraints on access. Future work should combine repeated sampling with concentration strategies (e.g., filtration and enrichment) to increase sensitivity and improve the utility of metagenomic environmental testing for source attribution.

Our offline analysis implemented rapid on-site mapping using KMA against a reduced-size bacterial reference database to enable fast processing on a laptop, followed by more comprehensive mapping for validation. This multilayered approach aligns with the reality of outbreak response, where fast initial answers are needed for awareness, while deeper analyses can follow once time and compute allow. Such designs are increasingly recognized as critical to the successful integration of portable genomics into routine surveillance and response workflows.

Antimicrobial resistance signals were also detected (offline) across sites, including genes associated with multiple antibiotic classes. Genotype-based resistance prediction from stool metagenomes can provide early warning of resistance genes circulating in the local gut microbial reservoir. In cholera management, rehydration remains primary, [22] but antibiotics may be used for severe cases and to reduce duration and shedding; therefore, resistance surveillance remains relevant to both clinical guidance and public-health planning. Our findings also show that a substantial fraction of cholera-suspected cases were dominated by other bacterial pathogens, e.g., diarrheagenic *E. coli*, for which antimicrobial treatment is more commonly considered. In this, rapid AMR screening adds additional clinical and public-health value by providing early situational awareness of resistance determinants circulating among gastrointestinal pathogens beyond *V. cholerae*.

Future work should focus on our identified limitations during our on-site investigation. First, clinical metadata were minimal and we did not perform a prospective detailed comparison against serotype testing and culture for all samples; future evaluations should quantify sensitivity/specificity and formalize interpretation thresholds for *V. cholerae* and key GI pathogens in field conditions. Second, environmental sampling was limited due to accessibility and should be expanded with repeated sampling and concentration to enable inferences about potential sources. Other limitations of read-based metagenomic workflows include the inability to attribute the AMR genes to specific pathogens. It is to a degree possible by building Metagenomic-assembled genomes (MAGs), however, this is not feasible in a rapid frontline diagnostic workflow. The workflow is not designed or optimised for detecting RNA viruses, and viral pathogens therefore cannot be detected or excluded as the causative agent. Additionally, the time-bounded, site-based recruitment and small sample size may introduce selection bias and do not support population-level inference. No formal epidemiological sample-size or power calculation was performed because the study was designed as a field proof-of-concept rather than to estimate diagnostic accuracy, prevalence or population-level aetiology.

A recent costing study of comparable ONT workflow (extraction and library-preparation kits differed from our study) [68] estimated consumables costs of approximately €72 per sample at 12-plex and €39 per sample at 24-plex, including DNA extraction but excluding VAT, personnel, equipment and infrastructure, with the flowcell accounting for most of the consumable cost. This is however only an indicative cost benchmark, as it would require measurement of personnel time, equipment cost and depreciation, transport, shipping and local supply-chain costs, which were not collected for our study and may vary substantially between settings.

Our results support mobile, offline ONT metagenomic sequencing as a feasible frontline diagnostic approach for cholera-suspected gastrointestinal disease in resource-limited settings without access to grid power, internet, or centralised laboratory infrastructure. Within existing cholera response systems, this workflow is intended as a complementary escalation tool, not a replacement for routine diagnostics, especially that ONT diagnostic is more costly compared to traditional tests. But it can solve issues regarding early outbreak confirmation, investigation of negative or inconclusive rapid-test results and unusual clusters, and especially rapid response to unknown agents where rapid antigen tests are negative. By enabling rapid, culture-independent detection of *V. cholerae* alongside alternative aetiologies and AMR signals, this workflow can strengthen outbreak confirmation, improve diagnostic specificity at the point of care and front-line responses, and support timely public-health intervention where conventional diagnostics are delayed or unavailable.

## Supporting information

S1 Table Minimal metadata for the three sites. (DOCX)

S2 Table Metagenomic sequencing output summary. (DOCX)

S3 Table Summary table of all samples sequenced, including culturing, front-line, and HPC-analyses results. The final column includes the diagnostic pathogen call.
(XLSX)

S4 Table Most abundant bacterial species identified in the gut microbiome of individuals from Rugombo Health Centre. The results are based on the read-alignment to the comprehensive bacterial genomic database.
(DOCX)

S5 Table Most abundant bacterial species in the environmental samples from the Mparambo neighbourhood in Rugombo. (DOCX)

S6 Table Unique bacterial genera identified at each location. (XLSX)

S7 Table A 2x2 comparison table of metagenomic and culturing results on *V. cholerae* detection. The numbers reflect the number of samples.
(DOCX)

S8 Table Alignment to the cholerae-specific database. The 11 cholerae-positive samples already identified with frontline-diagnostics as well as culturing had sequences aligning to *V. cholerae*. O1 was the most dominant one in all samples. Sample R24 had a mix of O1 and the isolated nonO1/nonO139 originating from the same sample.
(DOCX)

S9 Table Most abundant bacterial species identified with read-mapping to a comprehensive bacterial genomic database in patients from Rutana District Hospital. (DOCX)

S10 Table Most abundant bacterial species identified with read-mapping to the comprehensive bacterial genomic database in individuals from Cishemere refugee camp. CLR values are calculated within each group (asymptomatic/symptomatic).
(DOCX)

S1 Fig STARD-like diagram showing the analysis of the faecal samples collected at each site. (DOCX)

S2 Fig Bacterial species differences between asymptomatic companions, cholerae-positive, cholerae-negative, and environmental samples from Rugombo Health Centre and Mparambo neighborhood in Rugombo. The analysis is based on the alignment to the comprehensive bacterial database. A) PCA clustering of the samples based on the bacterial species. The latrine sample clusters more closely to the companion samples, and the lake and open drain sample clusters within the patient samples. B) Comparison of the diversity measures of the cholerae-positive patient, cholerae-negative patient and asymptomatic companion samples.
(DOCX)

S3 Fig Overview of the metagenomic frontline diagnostic results. Positive detection of pathogens are colored red. Positive detection and AMR gene is colored blue. A) Metagenomic diagnostic results from Rutana District Hospital B) Metagenomic diagnostic results from the patients and companions from Rugombo Health Centre, as well as the three environmental samples from the Mparambo neighborhood in Rugombo C) Metagenomic diagnostic results from individuals with stomach discomfort and asymptomatic individuals from Cishemere refugee camp in Cibitoke.
(DOCX)

S4 Fig PCA clustering of the samples from Cishemere refugee camp based on the bacterial species. The samples are colored with blue being samples from the asymptomatic individuals, and red being from the symptomatic individuals.
(DOCX)

S5 Fig Virus composition across the tree study sites shown as normalised abundance calculated by adjusting the basepair counts aligning to the reference by the reference length and sequencing depth (similar to FPKM but with bp instead of fragment counts). If pathogens were detected using frontline metagenomics, it is indicated above the heatmap. A) Samples from Rugombo Health Centre. B) Samples from Rutana District Hospital. C) Samples from Cishemere refugee camp in Cibitoke.
(DOCX)

S6 Fig Phylogenetic tree based on core SNPs. The tree was constructed with 89% reference genome coverage (reference genome *Vibrio cholerae* N16961 with accession no. NZ_CP028827.1). A) Matrix of SNP pair counts. B) SNP tree.
(DOCX)

S7 Fig Linear association between the abundance of bacteria and viruses tested with Pearson’s correlation coefficient. A) *V. Cholerae* phages and *Vibrio cholerae*. B) *E. coli* phages and *E. coli*.
(DOCX)
