# Supplementary material for: Syndromic cholera diagnosis masks diverse causes of diarrhoeal disease in Burundi revealed by portable metagenomics
Source: PLoS Negl Trop Dis. 2026 Sep 1;20(9):e0014175. doi: 10.1371/journal.pntd.0014175 (PMC13557499; doi:10.1371/journal.pntd.0014175)
Supplement: Supplementary file 1 [file pntd.0014175.s018.pdf]

19th June 2026

Dear Jeffrey (Editor),

Re: Reviewer report for the manuscript PNTD-D-26-00557, titled, "Syndromic cholera diagnosis masks diverse causes of diarrhoeal disease in Burundi revealed by portable metagenomics."

Thanks a lot for giving me this wonderful opportunity to review the manuscript titled as above on cholera. I found this paper interesting to read. However, there are major issues with this current version that affect interpretation of the manuscript content. Please find my comments below for your action. I am available to provide any other required information and clarification.

Thank you so much again.

Yours sincerely,

Dr. Godfrey Bwire, MBChB, MPH, PhD

General comments

The authors Jensen et al, carried out a study in which they evaluated a mobile, culture-independent metagenomic sequencing workflow for on-site detection of gastrointestinal pathogens directly from faecal samples collected from three health facilities in Burundi. The facilities were one (1) health centre, one (1) hospital and one (1) refugee transit camp. Furthermore, the authors state that they also collected one Stool samples and environmental samples were collected and analysed from samples from one (1) communal latrine, from one (1) open drain and one (1) river. The authors found out that *V. cholerae* signals were only detected in a subset of suspected cholera cases, while many samples were dominated by alternative bacterial taxa. The authors concluded that Mobile, offline metagenomic sequencing enables rapid frontline characterization of gastrointestinal disease, especially cholera-suspected. This is an interesting finding that has potential to attract readers and policy markers especially those in resource limited

countries such as those in sub-Saharan Africa. However, there are major flaws with the study methods that affects its usability and replicability. Specific issues are below.

Major issues

1. Page 6, lines 145-161, Materials and Methods: Sample collection and study design. The authors include a sub-heading titled "Study design" yet they do not specify the epidemiological design that underpins this work. The type of design such as cross-sectional, cohort or case-control is not stated, and other essential elements required for replication are missing such as sample size and how it was derived, sampling frame, inclusion and exclusion criteria, sampling method. Without a clearly defined design, the study does not meet basic reporting standards such as STROBE Item 4 and ICMJE recommendations, which require explicit description of the design to allow other scientists to repeat the study under comparable conditions.

2. Page 5, lines 120–123: "In Burundi, cholera (Vibrio cholerae) is considered an endemic disease, but with occasional outbreaks… inadequate sanitation, limited access to clean drinking water, and natural disasters." The authors correctly state cholera is endemic in Burundi hence suspected cases should be frequently seen. Page 6, lines 152–157: The authors report investigating only 31 suspected cholera cases at Rugombo Health Centre but do not state the target population, sampling frame, or selection method. Without this, it is unclear if the 31 cases represent complete enumeration or a non-random subset, which prevents assessment of selection bias. The authors should specify the sampling approach, case definition of a suspected cholera case, and rationale for 31 in Rugombo health centre or 9 in Rutana district being sufficient for their aims. Otherwise, the authors should limit conclusions to a descriptive report of these suspected cases only. The same explanation apply to the 21 cases investigated in Cishemere refugee transit centre district hospital.

3. Page 7, lines 145–168, Materials and Methods: The authors report samples from Rutana District 9, Cishemere Refugee Transit Camp 21, Rugombo Health Centre 36, and n=1 each from Rugombo open drainage, latrine, and river. The rationale for selecting only one site or facility per environmental source is not stated. Latrines, drainage points, and rivers occur as multiple units per community, and one health

centre cannot represent district care-seeking. With n=1 per environmental source there is no variance, so results lack precision and external validity. Unequal allocation without a stated sampling frame introduces selection bias and precludes valid clinical vs environmental comparisons. The authors must define the target population, sampling frame, selection method, and justification for site-specific sample sizes. It is not clear why the authors preferred n=1 in terms of sites and environmental samples. Conclusions should therefore be limited to descriptive reporting of the specific units investigated.

4. Page 4, lines 101–104, Introduction: The authors state: "To address these diagnostic constraints, there is growing interest in approaches that can provide rapid, culture-independent identification of gastrointestinal infection agents, including V. cholerae, directly from clinical samples, while remaining deployable in resource-limited and outbreak-prone settings." This frames the study as validating a rapid, culture-independent method for resource-limited settings. However, the manuscript does not present data supporting suitability for such settings. There is no analysis of reagent, equipment, or operational costs, and no comparison to standard-of-care or gold-standard methods on cost, infrastructure, or turnaround time. The results instead report performance metrics that reflect general analytical validity, not resource constraints. Thus, the claim of applicability to resource-limited settings is unsupported by the data presented.

Other comments

5. Page 16–17, lines 384–394, Figure 4 and legend and lines 397–409: The figures are clear and the data are valid, but they are irrelevant to the study aim. The goal is a rapid diagnostic test for gastrointestinal infections that works in resource-limited settings. Detecting V. cholerae and other pathogens is valid data. Also CTXϕ confirmation shows the platform works in the lab, not that it is rapid or field-deployable. The authors do not compare to culture, the standard of care, and they report no sensitivity, specificity, positive or negative predictive value. There is also no data on cost per test. Hence, claims about resource-limited use should be removed until comparison to culture or Gold standard are provided.

6. Across the manuscript: The authors use "suspected cholera" and "gastrointestinal symptoms" throughout but never define them. Undefined case definitions introduce speculation and limit reproducibility. To avoid ambiguity the authors should specify diagnostic criteria for both terms.

END
