# Supplementary material for: Syndromic cholera diagnosis masks diverse causes of diarrhoeal disease in Burundi revealed by portable metagenomics
Source: PLoS Negl Trop Dis. 2026 Sep 1;20(9):e0014175. doi: 10.1371/journal.pntd.0014175 (PMC13557499; doi:10.1371/journal.pntd.0014175)
Supplement: Supplementary file 2 [file pntd.0014175.s001.docx]

**S1 Table**

**Rugombo Health Centre metadata**

| **Flowcell** | **Sample** | **Location** | **Collection date** | **Sex** | **Age** | **Health status** |
| --- | --- | --- | --- | --- | --- | --- |
| Rugombo 2 | R6 | Rugombo Health Centre | 16-09-2025 | F | 16 months | Symptomatic |
| Rugombo 2 | R9 | Rugombo Health Centre | 16-09-2025 | F | 10 years | Symptomatic |
| Rugombo 2 | R10 | Rugombo Health Centre | 16-09-2025 | F | 3.6 years | Symptomatic |
| Rugombo 1 | R12 | Rugombo Health Centre | 16-09-2025 | F | 28 months | Symptomatic |
| Rugombo 1 | R13 | Rugombo Health Centre | 16-09-2025 | F | 39 years | Symptomatic |
| Rugombo 1 | R14 | Rugombo Health Centre | 16-09-2025 | F | 25 years | Symptomatic |
| Rugombo 1 | R15 | Rugombo Health Centre | 16-09-2025 | F | 19 years | Symptomatic |
| Rugombo 2 | R17 | Rugombo Health Centre | 16-09-2025 |  | 34 months | Symptomatic |
| Rugombo 1 | R20 | Rugombo Health Centre | 16-09-2025 | M | 11 years | Symptomatic |
| Rugombo 1 | R22 | Rugombo Health Centre | 16-09-2025 | M | 4 years | Symptomatic |
| Rugombo 2 | R24 | Rugombo Health Centre | 16-09-2025 | M | 31 months | Symptomatic |
| Rugombo 1 | R26 | Rugombo Health Centre | 16-09-2025 | M | 12 years | Symptomatic |
| Rugombo 2 | R31 | Rugombo Health Centre | 16-09-2025 | M | 3 years | Symptomatic |
| Rugombo 1 | R32 | Rugombo Health Centre | 16-09-2025 | F | 40 years | Companion to R13 |
| Rugombo 1 | R33 | Rugombo Health Centre | 16-09-2025 | F | 38 years | Companion to R15 |
| Rugombo 2 | R34 | Rugombo Health Centre | 16-09-2025 | F | 15 years | Companion to R17 |
| Rugombo 1 | R35 | Rugombo Health Centre | 16-09-2025 |  |  | Companion to R37 |
| Rugombo 2 | R36 | Rugombo Health Centre | 16-09-2025 | F | 16 years | Companion to R10 |
| Rugombo 2 | OR_2 | Rugombo Health Centre | NA | NA | NA | Symptomatic |
| Rugombo 2 | OR_4 | Rugombo Health Centre | NA | NA | NA | Symptomatic |
| Rugombo 2 | OR_7 | Rugombo Health Centre | NA | NA | NA | Symptomatic |
| Rugombo 2 | OR_8 | Rugombo Health Centre | NA | NA | NA | Symptomatic |
| Rugombo 2 | OR_9 | Rugombo Health Centre | NA | NA | NA | Symptomatic |
| Rugombo 2 | OR_12 | Rugombo Health Centre | NA | NA | NA | Symptomatic |
| Rugombo 2 | OR_13 | Rugombo Health Centre | NA | NA | NA | Symptomatic |
| Rugombo 2 | OR_14 | Rugombo Health Centre | NA | NA | NA | Symptomatic |
| Rugombo 2 | OR_15 | Rugombo Health Centre | NA | NA | NA | Symptomatic |
| Rugombo 2 | OR_16 | Rugombo Health Centre | NA | NA | NA | Symptomatic |
| Rugombo 2 | OR_17 | Rugombo Health Centre | NA | NA | NA | Symptomatic |
| Rugombo 2 | OR_19 | Rugombo Health Centre | NA | NA | NA | Symptomatic |
| Rugombo 2 | OR_20 | Rugombo Health Centre | NA | NA | NA | Symptomatic |
| Rugombo 2 | OR_21 | Rugombo Health Centre | NA | NA | NA | Symptomatic |
| Rugombo 2 | OR_22 | Rugombo Health Centre | NA | NA | NA | Symptomatic |
| Rugombo 2 | OR_23 | Rugombo Health Centre | NA | NA | NA | Symptomatic |

**Mparambo neighborhood in Rugombo metadata**

| **Flowcell** | **Sample** | **Location** | **Collection date** | **Sample type** |
| --- | --- | --- | --- | --- |
| Rutana + env | RL1 | Mparambo in Rugombo | 16-09-2025 | Latrine |
| Rutana + env | RR1 | Mparambo in Rugombo | 16-09-2025 | River |
| Rutana + env | B1 | Mparambo in Rugombo | 16-09-2025 | Open drain |

**Cishemere refugee camp in Cibitoke metadata**

| **Flowcell** | **Sample** | **Location** | **Collection date** | **Sex** | **Age (years)** | **Health status** |
| --- | --- | --- | --- | --- | --- | --- |
| Cishemere 1 | C1 | Cishemere refugee camp | 15-09-2025 | F | 34 | Asymptomatic |
| Cishemere 1 | C3 | Cishemere refugee camp | 15-09-2025 | F | 43 | Asymptomatic |
| Cishemere 1 | C4 | Cishemere refugee camp | 15-09-2025 | F | 5 | Symptomatic |
| Cishemere 1 | C5 | Cishemere refugee camp | 15-09-2025 | M | 10 | Symptomatic |
| Cishemere 1 | C8 | Cishemere refugee camp | 15-09-2025 | M | 7 | Asymptomatic |
| Cishemere 1 | C9 | Cishemere refugee camp | 15-09-2025 | F | 9 | Asymptomatic |
| Cishemere 1 | C11 | Cishemere refugee camp | 15-09-2025 | M | 37 | Asymptomatic |
| Cishemere 1 | C12 | Cishemere refugee camp | 15-09-2025 | F | 40 | Asymptomatic |
| Cishemere 1 | C10 | Cishemere refugee camp | 15-09-2025 | F | 4 | Symptomatic |
| Cishemere 1 | C13 | Cishemere refugee camp | 15-09-2025 | M | 31 | Asymptomatic |
| Cishemere 2 | C18 | Cishemere refugee camp | 15-09-2025 | M | 0.58 | Symptomatic |
| Cishemere 2 | C26 | Cishemere refugee camp | 15-09-2025 | F | 24 | Symptomatic |
| Cishemere 2 | C31 | Cishemere refugee camp | 15-09-2025 | F | 10 | Symptomatic |
| Cishemere 2 | C21 | Cishemere refugee camp | 15-09-2025 | M | 9 | Symptomatic |
| Cishemere 2 | C20 | Cishemere refugee camp | 15-09-2025 | M | 5 | Symptomatic |
| Cishemere 2 | C22 | Cishemere refugee camp | 15-09-2025 | M | 12 | Asymptomatic |
| Cishemere 2 | C19 | Cishemere refugee camp | 15-09-2025 | F | 8 | Symptomatic |
| Cishemere 2 | C23 | Cishemere refugee camp | 15-09-2025 | F | 27 | Symptomatic |
| Cishemere 2 | C16 | Cishemere refugee camp | 15-09-2025 | F | 10 | Asymptomatic |
| Cishemere 2 | C34 | Cishemere refugee camp | 15-09-2025 | F | 35 | Symptomatic |
| Cishemere 2 | C25 | Cishemere refugee camp | 15-09-2025 | M | 1.75 | Symptomatic |

**Rutana District Hospital metadata**

| **Flowcell** | **Sample** | **Location** | **Collection date** | **Sex** | **Age** | **Symptoms** | **Faeces** | **Place of residence (province, commune, quarter)** | **Treatment drug** |
| --- | --- | --- | --- | --- | --- | --- | --- | --- | --- |
| Rutana + env | RH1 | Rutana District Hospital | 18-09-2025 |  | 14 months |  | Non-liquid | Burunga, Rutana, Murama | Ampicillin, artesunate |
| Rutana + env | RH2 | Rutana District Hospital | 18-09-2025 | F | 3 years | Fever |  | Burunga, Rutana, Bugiga | Ampicillin, artesunate |
| Rutana + env | RH3 | Rutana District Hospital | 18-09-2025 | F | 14 months | Diarrhoea | Liquid | Burunga (province), Rutana (commune), Kayove (quarter) | Ampicillin |
| Rutana + env | RH4 | Rutana District Hospital | 18-09-2025 | M | 5 months | Diarrhoea | Liquid | Burunga, Rutana, Matutu | Ampicillin |
| Rutana + env | RH5 | Rutana District Hospital | 18-09-2025 | M | 18 months | Diarrhoea | Liquid | Burunga, Rutana, Kinzanza | Ampicillin, zink |
| Rutana + env | RH6 | Rutana District Hospital | 18-09-2025 | F | 3 years | Fever, anemia | Non-liquid | Burunga, Rutana, Butovyi | Ampicillin, metronidazole |
| Rutana + env | RH7 | Rutana District Hospital | 18-09-2025 | F | 6 months | Diarrhoea | Non-liquid | Burunga, Rutana, Rongero | Ampicillin |
| Rutana + env | RH8 | Rutana District Hospital | 18-09-2025 | M | 8 years | Diarrhoea, fever |  | Burunga, Rutana, Bugiga | Metronidazole, albendazole |
| Rutana + env | RH9 | Rutana District Hospital | 18-09-2025 | M | 13 months | Fever |  | Burunga, Rutana, Rongero | Albendazole |
