# Supplementary material for: Syndromic cholera diagnosis masks diverse causes of diarrhoeal disease in Burundi revealed by portable metagenomics
Source: PLoS Negl Trop Dis. 2026 Sep 1;20(9):e0014175. doi: 10.1371/journal.pntd.0014175 (PMC13557499; doi:10.1371/journal.pntd.0014175)
Supplement: Supplementary file 3 [file pntd.0014175.s002.docx]

**S2 Table :** Metagenomic sequencing output summary.

| **Run** | **Basepairs** | **Reads** | **N50** | **Run time** | **Median quality** |
| --- | --- | --- | --- | --- | --- |
| Rugombo run 1 (12 samples) | 3.97 Gb | 1.2 M | 4.93 kb | 45h 7 min | 16.35 |
| Rugombo run 2 (22 samples) | 6.83 Gb | 3.2 M | 3.37 kb | 22h 40 min | 15.10 |
| Rutana District Hospital and Rugombo environmental samples | 2.28 Gb | 0.9 M | 5.26 kb | 24h 53 min | 13.65 |
| Cishemere in Cibitoke run 1 | 0.62 Gb | 0.3 M | 4.28 kb | 32h 33 min | 15.40 |
| Cishemere in Cibitoke run 2 | 5.55 Gb | 2.2 M | 5.07 kb | 19h 55 min | 15.50 |
