# Supplementary material for: Syndromic cholera diagnosis masks diverse causes of diarrhoeal disease in Burundi revealed by portable metagenomics
Source: PLoS Negl Trop Dis. 2026 Sep 1;20(9):e0014175. doi: 10.1371/journal.pntd.0014175 (PMC13557499; doi:10.1371/journal.pntd.0014175)
Supplement: Supplementary file 4 [file pntd.0014175.s004.docx]

**S4 Table** **:** Most abundant bacterial species identified in the gut microbiome of individuals from Rugombo Health Centre. The results are based on the read-alignment to the comprehensive bacterial genomic database.

| **Rugombo**  **asymptomatic**  **companions** | |  | **Rugombo**  **cholerae-positive**  **patients** | |  | **Rugombo**  **cholerae-negative**  **patients** | |
| --- | --- | --- | --- | --- | --- | --- | --- |
| **Bacterial species** | **CLR median** |  | **Bacterial species** | **CLR median** |  | **Bacterial species** | **CLR median** |
| *Klebsiella quasipneumoniae* | 13.613 |  | *Vibrio cholerae* | 16.702 |  | *Klebsiella quasipneumoniae* | *14.195* |
| *Segatella hominis* | 12.722 |  | *Klebsiella quasipneumoniae* | 14.952 |  | *Escherichia coli* | *13.556* |
| *Prevotella stercorea* | 11.322 |  | *Escherichia coli* | 14.833 |  | *Roseburia inulinivorans* | *9.432* |
| *Escherichia coli* | 11.025 |  | *Actinomadura cremea* | 10.465 |  | *Actinomadura cremea* | *9.184* |
| *Alcanivorax profundi* | 10.491 |  | *Prevotella stercorea* | 10.449 |  | *Prevotella stercorea* | *9.059* |
| *Bacteroides kribbi* | 10.078 |  | *Segatella hominis* | 9.871 |  | *Segatella hominis* | *7.218* |
| *Prevotella pectinovora* | 9.330 |  | *Streptomyces gancidicus* | 7.744 |  | *Prevotella pectinovora* | *3.761* |
| *Phascolarctobacterium succinatutens* | 9.015 |  | *Alcanivorax profundi* | 7.297 |  | *Streptomyces gancidicus* | *3.489* |
| *Ruminococcus callidus* | 8.067 |  | *Prevotella pectinovora* | 6.874 |  | *Alcanivorax profundi* | *3.098* |
| *Roseburia inulinivorans* | 8.067 |  |  |  |  | *Streptomyces djakartensis* | *2.803* |
| *Holdemanella porci* | 7.794 |  |  |  |  |  |  |
| *Catenibacterium mitsuokai* | 7.775 |  |  |  |  |  |  |
| *Blautia wexlerae* | 7.727 |  |  |  |  |  |  |
| *Butyrivibrio crossotus* | 7.248 |  |  |  |  |  |  |
| *Prevotellamassilia timonensis* | 7.186 |  |  |  |  |  |  |
| *Ruminococcus flavefaciens* | 7.168 |  |  |  |  |  |  |
| *Ruminobacter amylophilus* | 7.017 |  |  |  |  |  |  |
| *Prevotella colorans* | 6.778 |  |  |  |  |  |  |
| *Streptomyces djakartensis* | 6.585 |  |  |  |  |  |  |
| *Thalassococcus profundi* | 6.559 |  |  |  |  |  |  |
| *Blautia stercoris* | 6.242 |  |  |  |  |  |  |
| *Waltera intestinalis* | 6.185 |  |  |  |  |  |  |
| *Blautia glucerasea* | 6.151 |  |  |  |  |  |  |
| *Acetivibrio ethanolgignens* | 6.141 |  |  |  |  |  |  |
| *Streptomyces viridodiastaticus* | 6.098 |  |  |  |  |  |  |
| *Elizabethkingia argenteiflava* | 5.821 |  |  |  |  |  |  |
| *Gemmiger formicilis* | 5.554 |  |  |  |  |  |  |
| *Ruminococcus bromii* | 5.099 |  |  |  |  |  |  |
