# Supplementary material for: Syndromic cholera diagnosis masks diverse causes of diarrhoeal disease in Burundi revealed by portable metagenomics
Source: PLoS Negl Trop Dis. 2026 Sep 1;20(9):e0014175. doi: 10.1371/journal.pntd.0014175 (PMC13557499; doi:10.1371/journal.pntd.0014175)
Supplement: Supplementary file 5 [file pntd.0014175.s005.docx]

**S5 Table** : Most abundant bacterial species in the environmental samples from the Mparambo neighbourhood in Rugombo.

| **Open drain (RB1)** | |  | **Latrine** **(RL1)** | |  | **River (RR1)** | |
| --- | --- | --- | --- | --- | --- | --- | --- |
| **Bacterial species** | **CLR** |  | **Bacterial species** | **CLR** |  | **Bacterial species** | **CLR** |
| *Klebsiella quasipneumoniae* | 11.428 |  | *Treponema peruense* | 14.675 |  | *Klebsiella quasipneumoniae* | 11.773 |
| *Alcanivorax profundi* | 8.137 |  | *Segatella hominis* | 14.529 |  | *Escherichia coli* | 8.693 |
| *Segatella hominis* | 7.921 |  | *Clostridiales bacterium* | 13.824 |  |  |  |
|  |  |  | *Treponema berlinense* | 13.718 |  |  |  |
|  |  |  | *Klebsiella quasipneumoniae* | 13.657 |  |  |  |
|  |  |  | *Prevotella pectinovora* | 13.572 |  |  |  |
|  |  |  | *Leyella stercorea* | 11.753 |  |  |  |
|  |  |  | *Escherichia coli* | 11.547 |  |  |  |
|  |  |  | *Alcanivorax profundi* | 10.575 |  |  |  |
|  |  |  | *Bacteroides kribbi* | 9.941 |  |  |  |
|  |  |  | *Phascolarctobacterium succinatutens* | 9.376 |  |  |  |
|  |  |  | *Agathobaculum butyriciproducens* | 8.344 |  |  |  |
|  |  |  | *Blautia wexlerae* | 8.255 |  |  |  |
|  |  |  | *Prevotella colorans* | 7.974 |  |  |  |
|  |  |  | *Blautia stercoris* | 7.826 |  |  |  |
|  |  |  | *Phocea massiliensis* | 7.766 |  |  |  |
|  |  |  | *Victivallis vadensis* | 7.691 |  |  |  |
|  |  |  | *Mediterraneibacter faecis* | 7.615 |  |  |  |
|  |  |  | *Treponema porcinum* | 7.561 |  |  |  |
|  |  |  | *Ruminobacter amylophilus* | 7.524 |  |  |  |
|  |  |  | *Thalassococcus profundi* | 7.473 |  |  |  |
|  |  |  | *Ruminococcus bromii* | 7.400 |  |  |  |
|  |  |  | *Oliverpabstia intestinalis* | 7.303 |  |  |  |
|  |  |  | *Elizabethkingia argenteiflava* | 7.159 |  |  |  |
|  |  |  | *Sellimonas caecigallum* | 7.156 |  |  |  |
|  |  |  | *Gemmiger formicilis* | 7.114 |  |  |  |
|  |  |  | *Waltera intestinalis* | 7.002 |  |  |  |
|  |  |  | *Streptomyces longisporoflavus* | 6.884 |  |  |  |
|  |  |  | *Prevotellamassilia timonensis* | 6.832 |  |  |  |
|  |  |  | *Roseburia inulinivorans* | 6.786 |  |  |  |
