# Supplementary material for: Syndromic cholera diagnosis masks diverse causes of diarrhoeal disease in Burundi revealed by portable metagenomics
Source: PLoS Negl Trop Dis. 2026 Sep 1;20(9):e0014175. doi: 10.1371/journal.pntd.0014175 (PMC13557499; doi:10.1371/journal.pntd.0014175)
Supplement: Supplementary file 6 [file pntd.0014175.s007.docx]

**S7 Table**

**S7 Table :** A 2x2 comparison table of metagenomic and culturing results on *V. cholerae* detection*.* The numbers reflect the number of samples.

|  | **Presumptive TCBS culture positive** | **Presumptive TCBS culture negative** |
| --- | --- | --- |
| **Metagenomics positive** | 11 | 0 |
| **Metagenomics negative** | 0 | 20 |
