# Supplementary material for: Syndromic cholera diagnosis masks diverse causes of diarrhoeal disease in Burundi revealed by portable metagenomics
Source: PLoS Negl Trop Dis. 2026 Sep 1;20(9):e0014175. doi: 10.1371/journal.pntd.0014175 (PMC13557499; doi:10.1371/journal.pntd.0014175)
Supplement: Supplementary file 7 [file pntd.0014175.s008.docx]

**S8** **Table**

**S8** **Table:** Alignment to the cholerae-specific database. The 11 cholerae-positive samples already identified with frontline-diagnostics as well as culturing had sequences aligning to *V. cholerae*. O1 was the most dominant one in all samples. Sample R24 had a mix of O1 and the isolated nonO1/nonO139 originating from the same sample.

| **Sample** | ***V. cholerae* isolate** | **Basepairs aligning** |
| --- | --- | --- |
| OR_08 | ERS28937889_R15__O1__Africa__Burundi__2025 | 15823236 |
| OR_17 | ERS28937889_R15__O1__Africa__Burundi__2025  ERS28937892_R23__O1__Africa__Burundi__2025 | 16269778  5737712 |
| OR_21 | ERS28937889_R15__O1__Africa__Burundi__2025 | 11861370 |
| OR_23 | ERS28937889_R15__O1__Africa__Burundi__2025  ERS28937892_R23__O1__Africa__Burundi__2025  1450_Vc_2012EL_2176__O1__North_America__Haiti__2012 | 46112064  8416745  5018347 |
| R9 | ERS28937889_R15__O1__Africa__Burundi__2025  ERS28937892_R23__O1__Africa__Burundi__2025  1450_Vc_2012EL_2176__O1__North_America__Haiti__2012  1611_Vc_C6706__O1__South_America__Peru__1991  1704_Vc_CRC1106__O1__Asia__India__1962 | 174127807  26426211  15184759  5923095  5180554 |
| R10 | ERS28937889_R15__O1__Africa__Burundi__2025  ERS28937892_R23__O1__Africa__Burundi__2025  1450_Vc_2012EL_2176__O1__North_America__Haiti__2012 | 133763713  18334727  10140059 |
| R14 | ERS28937889_R15__O1__Africa__Burundi__2025  ERS28937892_R23__O1__Africa__Burundi__2025  1450_Vc_2012EL_2176__O1__North_America__Haiti__2012 | 72633157  9698182  6708963 |
| R15 | ERS28937889_R15__O1__Africa__Burundi__2025 | 30826548 |
| R22 | ERS28937889_R15__O1__Africa__Burundi__2025 | 6361387 |
| R23 | ERS28937889_R15__O1__Africa__Burundi__2025 | 23154475 |
| R24 | ERS28937889_R15__O1__Africa__Burundi__2025  ERS28937891_R24__non_O1_O139__Africa__Burundi__2025 | 39371201  9195828 |
