# Supplementary material for: Syndromic cholera diagnosis masks diverse causes of diarrhoeal disease in Burundi revealed by portable metagenomics
Source: PLoS Negl Trop Dis. 2026 Sep 1;20(9):e0014175. doi: 10.1371/journal.pntd.0014175 (PMC13557499; doi:10.1371/journal.pntd.0014175)
Supplement: Supplementary file 8 [file pntd.0014175.s009.docx]

**S9 Table**

**S9** **Table:** Most abundant bacterial species identified with read-mapping to a comprehensive bacterial genomic database in patients from Rutana District Hospital.

| **Patients from Rutana District Hospital** | |
| --- | --- |
| **Bacterial species** | **CLR** **median** |
| *Escherichia coli* | 14.630 |
| *Klebsiella quasipneumoniae* | 13.822 |
| *Alcanivorax profundi* | 8.462 |
| *Segatella hominis* | 7.461 |
| *Actinomadura cremea* | 6.864 |
| *Leyella stercorea* | 6.699 |
| *Thalassococcus profundi* | 5.434 |
| *Prevotella pectinovora* | 4.940 |
| *Megasphaera micronuciformis* | 4.714 |
