# Supplementary material for: Syndromic cholera diagnosis masks diverse causes of diarrhoeal disease in Burundi revealed by portable metagenomics
Source: PLoS Negl Trop Dis. 2026 Sep 1;20(9):e0014175. doi: 10.1371/journal.pntd.0014175 (PMC13557499; doi:10.1371/journal.pntd.0014175)
Supplement: Supplementary file 9 [file pntd.0014175.s010.docx]

**S10** **Table**

**S10** **Table:** Most abundant bacterial species identified with read-mapping to the comprehensive bacterial genomic database in individuals from Cishemere refugee camp. CLR values are calculated within each group (asymptomatic/symptomatic)

| **Asymptomatic individuals** | |  | **Symptomatic individuals** | |
| --- | --- | --- | --- | --- |
| **Bacterial species** | **CLR**  **median** |  | **Bacterial species** | **CLR**  **median** |
| *Segatella hominis* | 12.437 |  | *Segatella hominis* | 12.849 |
| *Klebsiella quasipneumoniae* | 11.369 |  | *Klebsiella quasipneumoniae* | 12.517 |
| *Leyella stercorea* | 9.905 |  | *Leyella stercorea* | 12.270 |
| *Escherichia coli* | 9.216 |  | *Escherichia coli* | 10.094 |
| *Alcanivorax profundi* | 8.077 |  | *Prevotella pectinovora* | 8.841 |
| *Roseburia inulinivorans* | 8.015 |  | *Bacteroides kribbi* | 8.837 |
| *Prevotella pectinovora* | 7.303 |  | *Alcanivorax profundi* | 8.665 |
| *Bacteroides kribbi* | 7.249 |  | *Coprococcus phoceensis* | 6.694 |
| *Prevotella colorans* | 5.340 |  | *Prevotella copri* | 6.640 |
| *Elizabethkingia argenteiflava* | 5.260 |  | *Roseburia inulinivorans* | 6.593 |
| *Streptomyces djakartensis* | -0.924 |  | *Ruminococcus callidus* | 6.447 |
| *Actinomadura cremea* | -0.924 |  | *Thalassococcus profundi* | 6.154 |
| *Coprococcus phoceensis* | -0.924 |  | *Actinomadura cremea* | 6.134 |
| *Blautia wexlerae* | -0.924 |  | *Prevotellamassilia timonensis* | 5.402 |
| *Thalassococcus profundi* | -0.924 |  | *Prevotella colorans* | 5.085 |
| *Treponema berlinense* | -0.924 |  | *Elizabethkingia argenteiflava* | 4.933 |
| *Roseburia porci* | -1.235 |  | *Blautia wexlerae* | 4.779 |
| *Ruminococcus bromii* | -1.235 |  | *Ruminococcus bromii* | 4.271 |
| *Alishewanella longhuensis* | -1.235 |  | *Streptomyces viridodiastaticus* | 2.995 |
| *Butyrivibrio crossotus* | -1.235 |  | *Agathobaculum butyriciproducens* | 2.277 |
