# Supplementary material for: Syndromic cholera diagnosis masks diverse causes of diarrhoeal disease in Burundi revealed by portable metagenomics
Source: PLoS Negl Trop Dis. 2026 Sep 1;20(9):e0014175. doi: 10.1371/journal.pntd.0014175 (PMC13557499; doi:10.1371/journal.pntd.0014175)
Supplement: Supplementary file 11 [file pntd.0014175.s012.docx]

**S2 Fig**

**S2 Fig** **:** Bacterial species differences between asymptomatic companions, cholerae-positive, cholerae-negative, and environmental samples from Rugombo Health Centre and Mparambo neighborhood in Rugombo. The analysis is based on the alignment to the comprehensive bacterial database. **A)** PCA clustering of the samples based on the bacterial species. The latrine sample clusters more closely to the companion samples, and the lake and open drain sample clusters within the patient samples. **B)** Comparison of the diversity measures of the cholerae-positive patient, cholerae-negative patient and asymptomatic companion samples.
