# Supplementary material for: Syndromic cholera diagnosis masks diverse causes of diarrhoeal disease in Burundi revealed by portable metagenomics
Source: PLoS Negl Trop Dis. 2026 Sep 1;20(9):e0014175. doi: 10.1371/journal.pntd.0014175 (PMC13557499; doi:10.1371/journal.pntd.0014175)
Supplement: Supplementary file 12 [file pntd.0014175.s013.docx]

**S3 Fig**

**S3 Fig :** Overview of the metagenomic frontline diagnostic results. Positive detection of pathogens are colored red. Positive detection and AMR gene is colored blue. **A)** Metagenomic diagnostic results from Rutana District Hospital **B)** Metagenomic diagnostic results from the patients and companions from Rugombo Health Centre, as well as the three environmental samples from the Mparambo neighborhood in Rugombo **C)** Metagenomic diagnostic results from individuals with stomach discomfort and asymptomatic individuals from Cishemere refugee camp in Cibitoke.
