# Supplementary material for: Syndromic cholera diagnosis masks diverse causes of diarrhoeal disease in Burundi revealed by portable metagenomics
Source: PLoS Negl Trop Dis. 2026 Sep 1;20(9):e0014175. doi: 10.1371/journal.pntd.0014175 (PMC13557499; doi:10.1371/journal.pntd.0014175)
Supplement: Supplementary file 13 [file pntd.0014175.s014.docx]

**S4 Fig**

**S4 Fig :** PCA clustering of the samples from Cishemere refugee camp based on the bacterial species. The samples are colored with blue being samples from the asymptomatic individuals, and red being from the symptomatic individuals.
