# Supplementary material for: Syndromic cholera diagnosis masks diverse causes of diarrhoeal disease in Burundi revealed by portable metagenomics
Source: PLoS Negl Trop Dis. 2026 Sep 1;20(9):e0014175. doi: 10.1371/journal.pntd.0014175 (PMC13557499; doi:10.1371/journal.pntd.0014175)
Supplement: Supplementary file 14 [file pntd.0014175.s015.docx]

**S5 Fig**

**S5 Fig** **:** Virus composition across the tree study sites shown as normalised abundance calculated by adjusting the basepair counts aligning to the reference by the reference length and sequencing depth (similar to FPKM but with bp instead of fragment counts). If pathogens were detected using frontline metagenomics, it is indicated above the heatmap. **A)** Samples from Rugombo Health Centre. **B)** Samples from Rutana District Hospital. **C)** Samples from Cishemere refugee camp in Cibitoke.
