# Supplementary material for: Syndromic cholera diagnosis masks diverse causes of diarrhoeal disease in Burundi revealed by portable metagenomics
Source: PLoS Negl Trop Dis. 2026 Sep 1;20(9):e0014175. doi: 10.1371/journal.pntd.0014175 (PMC13557499; doi:10.1371/journal.pntd.0014175)
Supplement: Supplementary file 15 [file pntd.0014175.s016.docx]

**S6 Fig**

**S6 Fig :** Phylogenetic tree based on core SNPs. The tree was constructed with 89% reference genome coverage (reference genome *Vibrio cholerae* N16961 with accession no. NZ_CP028827.1). **A)** Matrix of SNP pair counts. **B)** SNP tree.
