# Supplementary material for: Syndromic cholera diagnosis masks diverse causes of diarrhoeal disease in Burundi revealed by portable metagenomics
Source: PLoS Negl Trop Dis. 2026 Sep 1;20(9):e0014175. doi: 10.1371/journal.pntd.0014175 (PMC13557499; doi:10.1371/journal.pntd.0014175)
Supplement: Supplementary file 16 [file pntd.0014175.s017.docx]

**S7 Fig**

**S7 Fig:** Linear association between the abundance of bacteria and viruses tested with Pearson’s correlation coefficient. **A)** *V. Cholerae* phages and *Vibrio cholerae*. **B)** *E. coli* phages and *E. coli*.
