# Supplementary material for: Syndromic cholera diagnosis masks diverse causes of diarrhoeal disease in Burundi revealed by portable metagenomics
Source: PLoS Negl Trop Dis. 2026 Sep 1;20(9):e0014175. doi: 10.1371/journal.pntd.0014175 (PMC13557499; doi:10.1371/journal.pntd.0014175)
Supplement: Supplementary file 17 [file pntd.0014175.s019.docx]

“We thank the three reviewers for their constructive and valuable comments, they genuinely improved the manuscript.
Please find our point-by-point responses below each comment in Green colour. All line numbers are from the tracked-change version of the manuscript”

Additional Editor Comments (if provided):

The reviewers generally agreed that this is an important study and that there were positives in the manuscript. However, there were issues raised by two of the reviewers that should be addressed in the revised manuscript. Please respond point by point to their comments and submit this with your revised manuscript.

Journal Requirements:

If the reviewer comments include a recommendation to cite specific previously published works, please review and evaluate these publications to determine whether they are relevant and should be cited. There is no requirement to cite these works unless the editor has indicated otherwise.

1) Please ensure that the CRediT author contributions listed for every co-author are completed accurately and in full.

At this stage, the following Authors/Authors require contributions: Emilie Egholm Bruun Jensen, Néhémie NZOYIKORERA, Mirena Ivanova, Pimlapas Leekitcharoenphon, Marie Noelle UWINEZA, Idrissa Diawara, Joseph Nyandwi, Frank M. Aarestrup, and Saria Otani. Please ensure that the full contributions of each author are acknowledged in the "Add/Edit/Remove Authors" section of our submission form.

The list of CRediT author contributions may be found here: <https://journals.plos.org/plosntds/s/authorship#loc-author-contributions>

“We already have an author contribution section (Lines 721-727). But added those contributions in the submission form now as well.”

2) Please upload all main figures as separate Figure files in .tif or .eps format. For more information about how to convert and format your figure files please see our guidelines:

<https://journals.plos.org/plosntds/s/figures>

“We have submitted them separately in the first submission, also now we submitted them separately.”

3) We have noticed that you have uploaded Supporting Information files, but you have not included a list of legends. Please add a full list of legends for your Supporting Information files after the references list.

“We have added List of Legends to the SI file and at the end of the main manuscript.”

4) Please amend your detailed Financial Disclosure statement. This is published with the article. It must therefore be completed in full sentences and contain the exact wording you wish to be published.

1) State the initials, alongside each funding source, of each author to receive each grant. For example: "This work was supported by the National Institutes of Health (####### to AM; ###### to CJ) and the National Science Foundation (###### to AM)."

2) State what role the funders took in the study. If the funders had no role in your study, please state: "The funders had no role in study design, data collection and analysis, decision to publish, or preparation of the manuscript."

3) If any authors received a salary from any of your funders, please state which authors and which funders..

If you did not receive any funding for this study, please simply state: u201cThe authors received no specific funding for this work.u201d

“We have updated our funding section and added a Financial Disclosure section.”

5) We have amended your Competing Interest statement to comply with journal style. We kindly ask that you double check the statement and let us know if anything is incorrect.

“We agree with the amended statement.”

6) Thank you for stating "The sequencing data generated during the study is available through the European Nucleotide Archive (ENA) repository: PRJEB108124. This includes metagenomic sequencing reads as well as reads and assemblies of five V. cholerae single isolates, including the circularised non O1/O139 strain (R24). " However, we were unable to access the sequence data using the information provided. Please review and update the Data Availability Statement to ensure that the underlying data are publicly accessible and that all accession numbers, links, or repository details are correct and functional.

“Thank you for this. They are now made public.”

**Reviewers' Comments:**

Reviewer's Responses to Questions

Key Review Criteria Required for Acceptance?

As you describe the new analyses required for acceptance, please consider the following:

**Methods**

-Are the objectives of the study clearly articulated with a clear testable hypothesis stated?

-Is the study design appropriate to address the stated objectives?

-Is the population clearly described and appropriate for the hypothesis being tested?

-Is the sample size sufficient to ensure adequate power to address the hypothesis being tested?

-Were correct statistical analysis used to support conclusions?

-Are there concerns about ethical or regulatory requirements being met?

Reviewer #1: --In general, very clear and detailed.

--There are many places here where results are presented (line 152, 169-171, etc.). The results of how many cholera samples were taken belongs at the start of the results.

“These results were briefly mentioned in the methods for practical reasons to keep the flow, they have now been moved to the results section (LINES 359-360)”

--May be helpful to add as an appendix all of the bacterial genera that could be detected?

“We thank the reviewer for this. Now Table S6 is added to the manuscript with all the bacterial genera identified at each location.”

Reviewer #2: The manuscript addresses a crucial and timely public health question: whether portable, offline metagenomic sequencing can enhance etiologic resolution among patients with clinically suspected cholera in resource-limited settings. The rationale is strong because the authors clearly describe the limitations of syndromic cholera diagnosis and centralized laboratory confirmation in Burundi and similar settings. The study objective is broadly stated as evaluating a mobile, culture-independent metagenomic workflow for on-site detection of Vibrio cholerae and other gastrointestinal pathogens directly from fecal samples. However, the manuscript would be stronger if the authors explicitly stated a primary testable hypothesis and separated the primary objective from secondary objectives. For example, the primary objective could be framed as evaluating the feasibility and concordance of an offline ONT-based workflow against culture and comprehensive bioinformatic analysis, while secondary objectives could include the detection of alternative bacterial taxa, CTX, AMR genes, and environmental signals. The current objective is clear in general terms, but it is not yet framed as a formal hypothesis-driven or diagnostic evaluation study.

“We agree with the reviewer that it is hard for a reader to know which findings were primary. We framed the objective broadly because the study had a composite aim: to establish whether the workflow could be executed at all under field conditions, and simultaneously to see what it revealed. In practice this meant the feasibility and biological questions were entangled in the writing. We have now separated them explicitly. Primary objective: to determine whether a fully offline, laptop-based ONT metagenomic workflow can be executed to a pathogen and AMR call at the point of care without grid power or internet, and whether its frontline calls are concordant with conventional culture and with comprehensive HPC-based analysis of the same reads. Secondary objectives: to characterise alternative bacterial taxa in patients triaged as suspected cholera: to assess whether CTXφ co-detection supports discrimination of toxigenic signal; to describe AMR gene content; and to explore environmental signals. We have also added a sentence identifying the study type as a field feasibility and technology-evaluation study, so the analyses can be mapped onto a stated plan (LINES 143-155)”

The study design is appropriate as a field feasibility and proof-of-concept study, but it is not sufficiently powered or structured to support strong diagnostic accuracy, etiologic attribution, or public health implementation claims. The authors sampled 31 suspected cholera cases and five asymptomatic companions at Rugombo Health Centre, nine gastrointestinal-symptom patients at Rutana District Hospital, 12 individuals with stomach discomfort and nine asymptomatic individuals at Cishemere refugee camp, plus three environmental samples. This is a valuable field dataset, but the sampling appears opportunistic rather than consecutive or prospectively powered.

“We agree that the study was not built to estimate diagnostic accuracy, pathogen prevalence or population-level etiology, as these were not the objectives of this proof-of-concept field evaluation. However, we must clarify that the sampling was not opportunistic in the sense of samples being selected because of particular clinical characteristics or anticipated results. The sites and deployment periods were planned in advance as it is a huge field study in remote areas in Burundi (multiple practical difficulties of road conditions and feasibility etc…), and participants were recruited from the eligible individuals accessible during each time-limited field visit, subject to the availability of a faecal sample and the operational capacity of the mobile workflow at the site.

We now describe the approach more precisely as time-bounded, site-based availability sampling under real-world field constraints (LINES 165-172), and added that sample numbers reflect the patients and samples available during that deployment window rather than a selected subset. We have clarified this procedure in the Methods and have limited our conclusions to workflow feasibility and the samples investigated, without making population-level prevalence or diagnostic-accuracy claims, those are modified in (e.g., LINES 659-663)”

The authors should clarify inclusion and exclusion criteria, whether all eligible patients during the sampling period were enrolled, how “suspected cholera,” “gastrointestinal symptoms,” and “stomach discomfort” were defined, and whether rapid diagnostic test, microscopy, culture, serology, PCR, dehydration status, stool appearance, antibiotic exposure, age, sex, symptom duration, and treatment information were available.

“We agree that the clinical categories and participant-identification procedures required clearer definitions. We have therefore revised the Methods to define each participant group according to how individuals were identified as following:

“patients with suspected cholera” refers to individuals classified as suspected cholera cases by the attending healthcare professionals and managed through the Cholera Treatment Centre during the field-deployment period. This was a routine clinical classification made independently of our arrival and before sequencing results were available (i.e., they were already identified by a health care professional there). We have now added this in the text.

“patients with gastrointestinal symptoms” refers to individuals identified by the attending clinical staff as presenting with a gastrointestinal complaint or illness. This was a broader clinical category than suspected cholera and was not intended to represent a single pathogen or illness. The specific clinical variables available for these patients varied, and were not systematically recorded and were identified as unavailable.
“stomach discomfort” refers to self-reported abdominal pain or discomfort at the time of sampling. These individuals did not report diarrhoea, and the collected stools were compact rather than watery. “Participants without reported gastrointestinal symptoms” refers to individuals who did not report stomach discomfort, abdominal pain, diarrhoea, vomiting or other gastrointestinal symptoms at the time of sampling.

All of the above has been adjusted to reflect the mentioned categories in the text (Lines 175-180, 213-224).

Participant recruitment was time-bounded and site-based during predefined field deployments. The categories were assigned before metagenomic analysis, and samples were not selected according to anticipated pathogen status or subsequent sequencing findings. We have clarified the recruitment periods, availability of faecal samples, inclusion procedures and limitations of the clinical metadata in the revised Methods (Several lines in the method section). No patients with symptoms were excluded from the study if they were present during the time we were there.”

The sample size is sufficient for a pilot feasibility demonstration but not sufficient for estimating diagnostic performance, pathogen prevalence, or etiologic fractions with precision. No sample size justification or power calculation is provided. This is acceptable if the study is explicitly presented as a field feasibility study, but the authors should avoid language implying definitive diagnostic accuracy or generalizable epidemiologic conclusions.

“We agree that the sample size does not support precise estimates of diagnostic accuracy, pathogen prevalence or population-level etiological fractions. These were not the objectives of the study. No formal power calculation was performed because the work was a time-limited field feasibility and proof-of-concept evaluation rather than a diagnostic-accuracy or epidemiological prevalence study. The number of samples was determined by the samples and participants available during the deployments, operational capacity and flowcell availability, cold chain maintenance amongst other practical requirements.

We now state this explicitly in the Methods and limitations and have removed language implying population representativeness or elaborate diagnostic performance (e.g., LINES 165-172 – Limitation section lines 648-663).”

The laboratory and bioinformatic methods are promising and mostly described in useful detail, including DNA extraction, ONT library preparation, flow cells, basecalling, demultiplexing, host-read removal, reduced-database KMA mapping, AMR screening, culture confirmation, isolate sequencing, and comprehensive HPC-based analyses. However, several methodological clarifications are required for acceptance. First, the authors should provide explicit pathogen-calling thresholds for the frontline workflow and justify the very permissive KMA identity threshold of 25% and minimum depth of 1.

“We now distinguish the parameters used to retain candidate mappings from the criteria used to interpret a reportable pathogen detection.

The frontline workflow was deliberately implemented as a two-stage analytical procedure. In the first stage, KMA was run against the reduced bacterial database using permissive output-retention settings (-ID 25 and -md 1). These settings were intended to retain candidate mappings for rapid on-site review and were not considered sufficient evidence of pathogen presence (we discuss the pathogenicity elsewhere). This approach follows previously published work on long-read KMA interpretation, including Gand et al. (2024) (<https://www.frontiersin.org/journals/microbiology/articles/10.3389/fmicb.2024.1336532/full> ) and others (<https://www.frontiersin.org/journals/microbiology/articles/10.3389/fmicb.2025.1597804/full> and <https://link.springer.com/article/10.1186/s42523-026-00577-2>) in which the minimum template-identity parameter was kept loose and the resulting output was subsequently interpreted using combinations of template identity, depth and template length. Importantly, in these studies, bacterial taxa assignments were confirmed using traditional methods when employing such thresholds. This avoids discarding potentially informative mappings at the initial computational stage while allowing weak or ambiguous mappings to be classified as low-confidence results requiring further evaluation.
KMA also uses ConClave scoring to resolve reads that map equally well to multiple templates in a redundant database. We have now clarified, however, that ConClave resolves competition among reference templates and does not itself constitute a pathogen-detection threshold (LINES 243-258).

In the second stage of our workflow, candidate frontline signals were evaluated using the comprehensive bacterial database, including a template breadth-of-coverage requirement of >50%. For *Vibrio cholerae*, the interpretation was additionally evaluated using the *V. cholerae*-specific reference database and the available culture and isolate-sequencing evidence. CTXφ co-detection was treated as supporting biological context regarding toxigenic potential and not as an independent confirmation of species detection.

We have now revised the Methods to distinguish explicitly between: (i) KMA mapping and candidate-retention parameters; (ii) the criteria used to classify a frontline signal as reportable; and (iii) the evidence used for subsequent comprehensive evaluation.

(LINES 243-258)”

Second, they should present concordance between the reduced laptop database and the full HPC database, ideally as a per-sample comparison table with agreement, discordance, and reasons for discordance.

“We thank the reviewer for this suggestion. A sample level comparison between the reduced laptop database and the comprehensive HPC database is already presented visually in Figure 3 for the Rugombo samples and Figure 4 for the Rutana and Cishemere samples. We agree, however, that the figures did not allow the reader to assess the agreement and reasons for any differences as readily as a table. We have therefore added Table S3, which reports the call from each workflow, the agreement status and, for any non-concordant sample, the most likely analytical explanation supported by the data. These explanations include differences in reference representation and taxonomic resolution resulting from the within-species strain collapsing used to construct the reduced database, or a low-abundance signal that did not meet the template-coverage criterion applied in the comprehensive analysis. Additional taxa detected by the comprehensive workflow were not classified as discordant when the frontline call was reproduced. We describe this as analytical concordance between two bioinformatic workflows and not as clinical sensitivity or specificity. We refer to Table S3 throughout the manuscript for such comparison.”

Third, if culture was used as confirmation for Rugombo samples, a simple 2×2 table comparing metagenomics and culture for V. cholerae should be provided, with exact binomial confidence intervals where appropriate. If culture, Sero grouping, or PCR were not performed for all samples, this limitation should be explicitly stated.

“We thank the reviewer and agree that the relationship between the metagenomic and culture-based findings should be stated in more detail. We have therefore added all the culturing results to the newly added Table S3, which presents, for each Rugombo sample, the frontline metagenomic result, comprehensive metagenomic result, presumptive TCBS culture results, colony-derived sequencing result, isolate purity, serogroup and sequence type where available.

All 11 samples containing high-abundance *V. cholerae* signals in both the frontline and comprehensive metagenomic analyses also produced yellow colonies following alkaline peptone water enrichment and culture on TCBS agar. Culture-derived material was sequenced for 10 of these 11 samples. Five yielded pure *V. cholerae* genomes, while the other five yielded mixed culture-derived sequence datasets in which *V. cholerae* was the predominant taxon. These ten samples are therefore described as having sequencing-supported culture recovery of *V. cholerae*, although only the five pure isolates were used for isolate-level genomic and serogroup characterisation as they have a higher bacterial coverage. The remaining sample, OR_17, produced yellow TCBS colonies but was not sequenced as the DNA was too degraded on-site and could not be used, and is therefore reported as presumptive culture-positive rather than sequencing-confirmed. All of this is already mentioned in the main manuscript (Lines 443-460).

All 31 Rugombo samples underwent the same culture procedure and that both positive and negative culture outcomes were systematically recorded, we have now also added a 2 × 2 comparison and report overall, positive and negative percent agreement (Table S7). We use agreement measures rather than sensitivity and specificity because neither analytical procedure was treated as a clinical reference standard.

Consistent with the reporting principle in STARD Item 11, we have also clarified the rationale and limitations of the available culture-based comparator (All method section). Enrichment followed by TCBS culture was the operationally available field procedure and allowed recovery of viable organisms for downstream characterisation. However, TCBS colony morphology is presumptive rather than species-specific, culture detects only organisms that remain culturable under the applied conditions, and colony recovery may be affected by factors such as selective growth, competing faecal organisms and prior antimicrobial exposure. In our study, the variable purity of culture-derived sequence datasets directly illustrates this limitation. Serogrouping and independent PCR confirmation were not performed systematically for every sample, and only serotyping is considered portable and on-site diagnostics (Serotype detection output is now in Table S3).

We have therefore avoided referring to culture as a definitive “gold standard.” The revised manuscript distinguishes presumptive TCBS growth, sequencing-supported recovery of *V. cholerae*, recovery of a pure isolate, and serogroup/sequence-type characterisation (Table 1, Table S3, Lines 443-460). We also state that the small sample size produces wide confidence intervals and that this exploratory agreement analysis does not constitute a formal diagnostic-accuracy evaluation (LINES 438-440)”

Statistical analysis requires strengthening. The use of alpha diversity, CLR transformation, PCA, ALDEx2, Welch’s tests with FDR correction, and Pearson correlations is generally reasonable for exploratory microbiome analysis; however, the manuscript would benefit from a clearer distinction between descriptive, exploratory, and confirmatory analyses. The authors should report effect estimates with uncertainty intervals wherever possible. The CTXφ correlation should include the number of samples included, p-value, confidence interval, sensitivity to zeros/outliers, and ideally a Spearman or permutation-based sensitivity analysis. Differential abundance testing among very small groups should be presented cautiously and should include sample sizes per comparison.

“We now distinguish between descriptive, exploratory and confirmatory analyses. All microbiome diversity and phage-correlation analyses are now explicitly described as exploratory (e.g., LINES 280-298). Sample sizes are reported for every comparison, effect estimates are presented where appropriate, and inferential language has been moderated.

For the CTXφ analysis, we now report the number of samples, Pearson coefficient, p-value and confidence interval, together with Spearman rank correlation and analyses (Figure S5A). Differential-abundance findings has been removed from the manuscript and are no longer used to support generalisable biological conclusions.”

A major concern is the interpretation of E. coli. The manuscript frequently refers to E. coli as a potential pathogen based on its abundance. However, E. coli is also a common gut commensal, and metagenomic species-level detection alone is insufficient to infer diarrheagenic E. coli. The authors should screen for and report diarrheagenic E. coli virulence markers or pathotype-associated genes, such as stx1/stx2, eae, bfpA, elt, est, ipaH, aggR, and related markers. Without these analyses, the manuscript should avoid implying that E. coli abundance alone indicates causation.

“We agree that abundance cannot infer pathogenicity alone and should be supported by virulence markers. To address this concern, we performed and added an additional analysis screening the metagenomic reads for established diarrhoeagenic E. coli virulence markers (Lines 294-298). Reads were mapped using KMA against the VFDB core dataset (24 July 2026). We screened for primary pathotype-associated markers, including stx1, stx2, eae, bfpA, eltA, eltB, est, ipaH and aggR, together with supporting markers including ehxA, saa, aatA, aaiC, tir and escV.

Diarrhoeagenic *E. coli* virulence markers were detected in 11 of the 15 samples containing high-abundance *E. coli* signals. We have added these results to Figure 3 and Table S3, which reports the individual genes, identity, coverage, depth and sample-level marker combinations (Lines 366-377).

We have also revised the interpretation to avoid assigning pathotypes more specifically than supported by the marker combinations. ipaH was interpreted as a Shigella/EIEC-associated marker because it cannot independently distinguish EIEC from Shigella. Detection of eae, escV and/or tir was described as a LEE-associated signature, an EPEC-compatible interpretation was made only where stx1/stx2 were absent, while detection of Shiga-toxin genes would support a STEC profile. The enterotoxin genes eltA/eltB and estIa were interpreted as ETEC-associated, while aatA alone was reported as an EAEC signal rather than definitive EAEC identification.

Because this analysis was performed on metagenomic material, marker detection was interpreted at the sample level, and not automatically taken as proof that all markers originated from the dominant *E. coli* population or from a single strain. We have therefore replaced statements that *E. coli* abundance “confirmed pathogenicity” with the more precise conclusion that virulence-marker detection provided evidence of diarrhoeagenic *E. coli* potential. We also avoid attributing disease causation solely from these metagenomic findings (Lines 468-471, 484-487).”

AMR analyses also require more detail. The authors should report gene-level AMR findings, identity and coverage thresholds, whether genes were assembled or read-based only, and whether any AMR genes could be linked to specific taxa or contigs. Stool metagenomic AMR detection provides useful surveillance information but should not be interpreted as direct clinical susceptibility evidence unless validated.

“We have added the needed details to the AMR gene analysis in Supplementary Table S3 (gene, identity, coverage, depth), also in the text (LINES 410-415, 474-476), which reports each detected ARG and allele, antimicrobial class, percentage identity, template coverage, depth, sample. All alignment analyses are based on read-mapping to databases, see the method section. All of the resistome outputs are based on read-based analyses, as linking ARGs to assembled genomes requires building MAGs of the bacterial genomes from the samples, which is extremely time-consuming and thus contradictory to the idea of rapid on-site metagenomic analyses and outside the scope of this research.

However, we note that metagenomic ARG detection is not limited to population surveillance. Direct-from-sample sequencing has increasingly been evaluated for clinically relevant resistance detection and prediction, including resistance profiling from clinical urine and faecal samples (<https://www.frontiersin.org/journals/cellular-and-infection-microbiology/articles/10.3389/fcimb.2026.1759322/full> <https://pmc.ncbi.nlm.nih.gov/articles/PMC11358271/> ), including several of our previous work (<https://link.springer.com/article/10.1186/s42523-026-00577-2>, <https://www.frontiersin.org/journals/microbiology/articles/10.3389/fmicb.2024.1336532/full> ).

We agree that the interpretation must reflect the results available from this study. Here, the ARGs were detected in the total stool metagenome and were not systematically linked to a specific bacterial pathogen or compared with organism-specific phenotypic susceptibility testing. We therefore do not present these findings as direct isolate-level AST or as definitive evidence that a particular detected pathogen was resistant. Instead, we describe them as clinically relevant, early resistome information that can provide situational awareness, identify important resistance determinants and guide prioritisation for subsequent culture, organism linkage and phenotypic testing.

The ethics statement reports approval by the National Ethics Committee in Burundi and a waiver of consent, with samples collected by health professionals. This is acceptable in principle, but because the study includes symptomatic patients, asymptomatic companions, and a refugee transit camp population, the authors should provide more detail on the waiver rationale, de-identification, handling of vulnerable populations, and whether environmental sampling required separate permissions.

“We have expanded the Ethics section and added the details of the approval provided by the National Ethics Committee for the waiver of individual consent. (LINES 707-719). The approval included all sample collection for the GREAT-LIFE project (Global Health European and Developing Countries Clinical Trials Partnership 3 (Global Health EDCTP3) Joint Undertaking programme), which include acquiring biological materials and their genetic content for the purpose of GREAT-LIFE project only, which this study is under. The approval from the National Ethics Committee of Burundi, INSP (National Health Institute in Burundi) and the Ministry of Health in Burundi included a waiver of individual informed consent for the collection of faecal samples and access to the biological material used in this study as they were collected by health professional during the routine diagnostics or normal defecation. Faecal sample collection was non-invasive and was performed by local healthcare professionals on site. The same approved procedures applied to symptomatic patients, companions without reported gastrointestinal symptoms and participants at the refugee transit camp.

Samples were assigned a unique study code by the healthcare professionals before transfer to us (the researchers). The researchers received only coded samples and minimal clinical metadata, with no names or other direct personal identifiers. Environmental sampling was included within the approved study activities and did not require separate ethical approval. We have expanded the Ethics section accordingly.”

Reviewer #3: -Are the objectives of the study clearly articulated with a clear testable hypothesis stated? YES

-Is the study design appropriate to address the stated objectives? NO. STUDY DESIGN NOT GIVEN

“See our detailed reply in Reviewer 3 comment below where they wrote them at the end.”

-Is the population clearly described and appropriate for the hypothesis being tested? NO.

“See our detailed reply in Reviewer 3 comment below where they wrote them at the end.”

-Is the sample size sufficient to ensure adequate power to address the hypothesis being tested? NO. SEE THE DETAILED REPORT.

“See our detailed reply in Reviewer 3 comment below where they wrote them at the end.”

-Were correct statistical analysis used to support conclusions? NO.

“See our detailed reply in Reviewer 3 comment below where they wrote them at the end.”

-Are there concerns about ethical or regulatory requirements being met? NO

“See our detailed reply in Reviewer 3 comment below where they wrote them at the end.”

**Results**

-Does the analysis presented match the analysis plan?

-Are the results clearly and completely presented?

-Are the figures (Tables, Images) of sufficient quality for clarity?

Reviewer #1: --Curious why Bacteroides was determined to be non-pathogenic?

“We did not intend to classify all members of the genus *Bacteroides* as intrinsically non-pathogenic. Several *Bacteroides* species are common members of the healthy intestinal microbiota, although members of the genus may also act as opportunistic pathogens under particular conditions. However, from our previous work (<https://pubmed.ncbi.nlm.nih.gov/40164980/>, <https://pubmed.ncbi.nlm.nih.gov/42158361/>) and the nature of these specific samples, we speculated these are commensal ones. We have replaced “non-pathogenic” with “commensal-associated gut taxa that were not interpreted as candidate primary gastrointestinal pathogens in this dataset.” We now also make clear that this interpretation was based on taxonomic composition alone and does not exclude strain-specific or opportunistic pathogenic potential (lines 387-393).”

--Can the authors distinguish which of the bacteria the AMR genes were linked to? Some of these are likely plasmid mediated and not necessarily able to be linked.

“Not from this analysis. Linking AMR genes to the bacterial host using metagenomics sequencing requires constructing MAGs (Metanomic Assembled Genomes) which is beyond the first line scope and diagnostic purpose here.”

Reviewer #2: The results generally follow the stated field-evaluation approach, but because the manuscript does not clearly define a formal analysis plan, it is difficult to determine which results were primary versus exploratory. The sequencing output section is useful and transparent, but the authors should add a consolidated table showing per-sample read counts, human-read percentage, bacterial-read count, dominant taxon, pathogen call, culture result, CTX result, AMR summary, and whether the reduced and full databases agreed.

“We have added the needed details to Table S3 to more clearly provide an overview of the results per method (culturing, frontline, HPC). We have also added a final “pathogen call” column to summarise the findings per sample (Table S3.) ”

The main finding that V. cholerae was detected in 11 of 31 suspected cholera cases, while other samples were dominated by other taxa, especially E. coli, is important and relevant. However, the result should be presented with more diagnostic structure. The manuscript should clearly show how many suspected cholera cases were positive by frontline metagenomics, comprehensive metagenomics, culture, serogroup/serotype testing, and CTX detection. The current presentation is promising but requires clearer quantitative alignment between platforms and methods.

“Figure 3 already presented this information, but perhaps it was over crowded. We have now updated it to more clearly present the pathogen detection per method (culturing, frontline, HPC). The result of samples positive of *V. cholerae* are linked with a line. We have added the CTX result to the figure as well. Additionally, we have added all the needed details to Supplementary Table S3. The text was also adjusted accordingly (Lines 383-388 and Figure 3).”

The figures are informative but need improvement for clarity. Figure 1 helps show the workflow and field sites. Figure 2 provides useful, sequencing quality-control information. Figure 3 is central to the manuscript, but it is visually dense, and the sample labels, pathogen-call indicators, and relative-abundance bars are difficult to interpret quickly. Figure 4 is also useful but should be clarified with larger labels, clearer color palettes, and more explicit separation of symptomatic, asymptomatic, and hospital samples. The authors should consider adding a simpler diagnostic summary figure or heatmap that directly compares culture, frontline metagenomics, comprehensive metagenomics, CTX, and AMR findings.

“We completely agree. We have modified Figures 3 and 4, and we have moved additional graphs from these figures that are not relevant to the major results to the supplementary material to simplify them. We have also included a more clear labelling of the frontline and HPC-based analyses in Figure 4, as well as more clear labelling of the hospital and refugee camp samples. Additionally, we have added the *E. coli* virulence markers to support the pathogenicity statements in both figure 3 and 4.”

The CTX result is one of the strongest biological supports for the V. cholerae interpretation, but it is currently underdeveloped. The authors should present the CTX correlation as a main figure or expanded supplementary figure with sample labels, correlation statistics, p-values, confidence intervals, and sensitivity analyses. This would make the claim that CTX helps distinguish toxigenic signal from background detection much more convincing.

“We very much agree that the CTXφ results strongly support the *V. cholerae* interpretation. We have now incorporated the result in the main Figure 3 to support the pathogen call across all methods (culturing, frontline, HPC), as well as added a per-sample result in Supplementary Table 3. Correlation between CTXφ and *V. cholerae* abundance is supported by statistics in Supplementary Figure 5A.”

The AMR results are potentially valuable but currently too general. Reporting resistance classes alone is insufficient. The authors should provide gene-level AMR calls and avoid overinterpreting clinical implications unless phenotype or organism-specific linkage is available.

“Supplementary Figure 2 already includes the AMR genes found in each sample. We have included the gene-level AMR findings per sample result in Supplementary Table S3 as well, and in several places in the text (please see the AMR comment above). We have also included the genes in our results text. Regarding the clinical interpretation of our resistome data, we have addressed this in the above comment to the same reviewer (reviewer 2), as following: We note that metagenomic ARG detection is not limited to population surveillance. Direct-from-sample sequencing has increasingly been evaluated for clinically relevant resistance detection and prediction, including resistance profiling from clinical urine and faecal samples (https://www.frontiersin.org/journals/cellular-and-infection-microbiology/articles/10.3389/fcimb.2026.1759322/full https://pmc.ncbi.nlm.nih.gov/articles/PMC11358271/ ), including several of our previous work (<https://link.springer.com/article/10.1186/s42523-026-00577-2> , <https://www.frontiersin.org/journals/microbiology/articles/10.3389/fmicb.2024.1336532/full> ).

We agree that the interpretation must reflect the results available from this study. Here, the ARGs were detected in the total stool metagenome and were not systematically linked to a specific bacterial pathogen or compared with organism-specific phenotypic susceptibility testing. We therefore do not present these findings as direct isolate-level AST or as definitive evidence that a particular detected pathogen was resistant. Instead, we describe them as clinically relevant, early resistome information that can provide situational awareness, identify important resistance determinants and guide prioritisation for subsequent culture, organism linkage and phenotypic testing.”

Reviewer #3: -Does the analysis presented match the analysis plan? NO

“See our detailed reply in Reviewer 3 comment below where they wrote them at the end.”

-Are the results clearly and completely presented? YES

-Are the figures (Tables, Images) of sufficient quality for clarity? YES

**Conclusions**

-Are the conclusions supported by the data presented?

-Are the limitations of analysis clearly described?

-Do the authors discuss how these data can be helpful to advance our understanding of the topic under study?

-Is public health relevance addressed?

Reviewer #1: --Very nice statement in lines 437-429.

--Very strong conclusions supported by the results.

“We thank the reviewer for finding it interesting.”

Reviewer #2: The core conclusion that portable offline metagenomics is feasible in field conditions and can provide broader etiologic insight than syndromic cholera diagnosis is supported by the data. The study is novel, operationally relevant, and well aligned with the public health needs of cholera-prone and resource-limited settings.

“We thank the reviewer for finding it novel and relevant.”

However, several conclusions should be moderated. The data support feasibility and descriptive detection, but not definitive diagnostic accuracy, pathogen causality, or clinical management impact. Claims about “alternative etiologies” should be softened unless virulence/pathotype evidence is added for organisms such as E. coli. Detection of a bacterial species in stool does not necessarily establish disease causation.

“We are aware that a bacterial species presence doesn’t mean causality of a disease. We have now tuned down this language when appropriate in the manuscript. However, we have also added a targeted virulence analysis for the *E. coli* members where we screened the samples for diarrheagenic *E. coli* virulence markers as suggested, to support the pathogenicity claim (Table S3, Figure 3, Figure 4). ”

The authors do acknowledge important limitations, including minimal clinical metadata, lack of a prospective detailed comparison against serotype testing and culture for all samples, and limited environmental sampling. These limitations are appropriate but should be expanded and moved into a more explicit limitations paragraph. Additional limitations should include small sample size, non-random/opportunistic sampling, lack of power calculation, limited controls, possible contamination or barcode crosstalk in multiplexed metagenomics, limited ability to detect RNA viruses if only DNA sequencing was performed, and inability to attribute AMR genes to specific pathogens without assembly/linkage evidence.

“We already have a section for limitation, it has now been expanded as well (LINES 648-663)”

The public health relevance is strong. The manuscript directly addresses outbreak response, delayed centralized diagnostics, and diagnostic uncertainty in suspected cholera cases. The authors should more clearly explain how this workflow fits into existing cholera response systems: for example, as an escalation tool when rapid tests are negative/inconclusive, during early outbreak confirmation, for sentinel surveillance, or for investigation of unusual clusters.

“We thank the reviewer for this positive comment, we have now added a sentence on how this workflow can be implemented, i.e., escalation when rapid tests are negative or inconclusive, early outbreak confirmation, sentinel testing at cholera treatment centres, and investigation of unusual clusters, and stating that it complements rather than replaces rapid tests and culture (LINES 673 onward)”

Reviewer #3: -Are the conclusions supported by the data presented? NO. SEE THE REPORT

“See our detailed reply in Reviewer 3 comment below where they wrote them at the end.”

-Are the limitations of analysis clearly described? NO. NOTHING ON THE COST YET THE STUDY IS RESOURCE LIMITED SETTING.

“See our detailed reply in Reviewer 3 comment below where they wrote them at the end.”

-Do the authors discuss how these data can be helpful to advance our understanding of the topic under study? YES

-Is public health relevance addressed? YES

**Editorial and Data Presentation Modifications?**

Use this section for editorial suggestions as well as relatively minor modifications of existing data that would enhance clarity. If the only modifications needed are minor and/or editorial, you may wish to recommend “Minor Revision” or “Accept”.

Reviewer #1: --Please be sure that genus/species names are italicized throughout.

“Those have been edited accordingly.”

--I would suggest avoiding the term "cholera-suspected diarrhea"; you could use acute watery diarrhea or another term that is more widely accepted and does not include the genus name.

“We agree, thank you. Those have been edited accordingly.”

--Patients are not "suspected of cholera" but instead they are patients "with suspected cholera."

“Those have been edited accordingly.”

--Line 93, is missing an "and" before time

“It has been added.”

--Line 120, should be "cholera" not cholerae when talking about the disease. The species is cholerae.

“This has been edited accordingly.”

--Line 292 and line 299 are missing a period at the end of the sentence after the parentheses.

“Those have been edited accordingly.”

Reviewer #2: Several editorial revisions are needed before acceptance.

First, the manuscript needs careful copyediting. Examples include “reply heavily” instead of “rely heavily,” inconsistent use of “cholerae” versus “cholera,” inconsistent spelling of “faecal/fecal” and “diarrhoeal/diarrheal,” “lap-top” versus “laptop,” and awkward phrases such as “sufficient high amount of reads” and “the absence of detections could be difficult.” The authors should also avoid referring to “cholerae-negative patients”; “V. cholerae-negative patients” would be clearer.

“All the above have been edited accordingly and throughout the manuscript”

Second, the authors should define all major abbreviations at first use, including CTX, ONT, KMA, AMR, CLR, HPC, TCBS, ABPW, ST, and FDR. The manuscript should also consistently italicize bacterial species names.

“Those have been edited accordingly.”

Third, the title is strong but slightly broad. Because the study is a pilot field evaluation rather than a definitive etiologic study, the authors may consider a more measured title such as: “Portable metagenomics reveals heterogeneous microbial profiles among suspected cholera cases in Burundi.”

“We thank the reviewer for this suggestion. We considered a more descriptive title along the lines proposed, however, after revising the manuscript to more clearly distinguish pathogen detection from definitive aetiological attribution, we have decided to retain the original title. We believe it more accurately captures the central finding of the study: that syndromic classification of patients with suspected cholera can encompass substantially different pathogen-associated profiles, which are revealed by onsite metagenomic analysis. The title is intended to reflect this diagnostic heterogeneity rather than to imply definitive causation, and we have moderated the corresponding language throughout the manuscript accordingly. ”

Fourth, the data presentation should be improved with the following additions: a STARD-like flow diagram of sample inclusion and testing, a per-sample diagnostic summary table, a table comparing reduced-database and full-database classifications, a table of AMR genes with identity/coverage thresholds, and a runtime/cost/power table for field deployment.

“We have now added a STARD-like sample flow diagram showing participant and sample inclusion and the analyses performed at each site (Figure S1). The new Table S3 now provides the per-sample diagnostic summary, reduced-versus-comprehensive database agreement, culture and CTXφ results, and gene-level AMR findings with identity and coverage information. As this was a field feasibility rather than a formal diagnostic-accuracy study, the diagram is used to improve reporting transparency and does not imply full STARD compliance.

We have added an indicative consumables cost benchmark to the Discussion (LINES 665-671). A recent costing study of a comparable ONT workflow estimated costs of approximately €72 per sample at 12-plex and €39 per sample at 24-plex, including DNA extraction but excluding VAT, personnel, equipment and infrastructure. The flowcell accounted for most of the consumable cost (<https://www.frontiersin.org/journals/public-health/articles/10.3389/fpubh.2026.1755877/full> ). Because the published workflow used different extraction and library-preparation kits, these figures are presented as contextual benchmarks rather than a direct costing of our field workflow.

We have deliberately not presented this as a cost effectiveness comparison with rapid tests or culture. Such an analysis would require measurement of personnel time, equipment cost and depreciation, transport, shipping and local supply-chain costs, which were not collected and may vary substantially between settings. We have also clarified that, in this manuscript, deployment in resource-limited settings refers specifically to operation without grid power, internet access or centralised laboratory infrastructure, as demonstrated in the study, rather than to lower per-sample cost, which we do not claim.”

The authors state that the workflow can generate actionable profiles in approximately 12 hours, but the sequencing durations reported range from about 11 to 45 hours; this discrepancy should be reconciled clearly.

“We thank the reviewer for raising this, it does seem ambiguous. The reported 11-45 hour durations refer to the total sequencing runs, whereas approximately 12 hours refers to the time required to obtain the initial frontline pathogen and AMR profile and the majority of the sequencing data. Because ONT reads were generated, basecalled and analysed continuously, the principal findings were available within approximately 12 hours for most runs, which also aligns with the nature of ONT sequencing as real-time sequencer. Sequencing was continued beyond this point (the 12 hours) to maximise read yield and improve the detection and characterisation of lower-abundance organisms (which are not the causative agents as the main pathogens are often abundant) and resistance determinants, but not because the initial result required the full sequencing duration. We have clarified this distinction and now report the time to the initial frontline result separately from the total sequencing duration (Lines 344-346).”

Fifth, the data availability statement is helpful and provides ENA accession. However, the authors should also provide scripts, pipeline parameters, reduced database construction details, database versioning, and ideally a reproducible workflow repository.

“ The reduced genomic database consists of bacterial genomes that have been collapsed into closely related strains. The installer is available from:

<https://bitbucket.org/genomicepidemiology/cgelabs-installer/src/main/>

We have provided scripts, including pipeline parameters, in the following GitHub repository:

<https://github.com/emilieegholmbruun/frontline_metagenomics.git>

These are added to the main text now.”

Reviewer #3: (No Response)

**Summary and General Comments**

Use this section to provide overall comments, discuss strengths/weaknesses of the study, novelty, significance, general execution and scholarship. You may also include additional comments for the author, including concerns about dual publication, research ethics, or publication ethics. If requesting major revision, please articulate the new experiments that are needed.

Reviewer #1: --I am unsure what the authors intend by this statement: "...can be infected by a wide range of other pathogens that are not detected by standard diagnostics." Do the authors mean standard CHOLERA diagnostics? Because other pathogens certainly can be detected or suspected based on simple diagnostic tests on stool including stool wbc, stool wbc, or O&P.

“We did mean cholera-specific diagnostics, rather than standard stool diagnostics more broadly. The sentence now reads: (Patients with suspected cholera may instead harbour other enteric pathogens that are not detected by cholera-specific diagnostic tests). (Lines 56-58)”

--Would also add to this sentence that another concern (besides dehydration) is easy transmission in places with substandard hygiene. "Because severe dehydration can develop within hours with diarrhoeal diseases, outbreak control and patient management depend on timely recognition of true cholera cases and differentiating them from other causes of gastrointestinal illness with overlapping symptoms."

“This has been edited accordingly to be (Lines 87-90) ”

--Would be good in the introduction to acknowledge co-infection of cholera + another bacterial pathogen also.

“We agree. These have been added now (Lines 103-105).”

--Very nice description in the introduction about the tests and Burundi, but please add a statement about aim/objective and the 3 populations that the test was deployed in.

“We have added an explicit statement of the study aim (LINES 143-147). Regarding the sites, we added the information that we were allowed to obtain due to GDPR.”

Reviewer #2: This is a timely, innovative, and potentially important manuscript that demonstrates the feasibility of deploying portable ONT metagenomics for diarrheal disease investigation in Burundi. The study’s major strengths include its field-based implementation, relevance to cholera outbreak response, inclusion of multiple clinical settings, integration of frontline laptop-based analysis with deeper HPC confirmation, and culture/WGS follow-up for selected V. cholerae isolates. The manuscript is especially valuable because it addresses a real operational problem: syndromic cholera diagnosis can be nonspecific, and delayed centralized testing can limit timely public health action.

“We thank the reviewer for finding it interesting and useful”

The main weaknesses are not conceptual but analytical and interpretive. The manuscript currently overstates some findings relative to the study design. It should be framed as a pilot feasibility and exploratory metagenomic field study rather than a fully validated diagnostic accuracy study. Required revisions include clearer objectives and hypotheses, stronger description of patient selection and clinical metadata, explicit pathogen-calling thresholds, diagnostic concordance tables, uncertainty estimates, improved CTX analysis, organism/pathotype-level interpretation of E. coli, gene-level AMR reporting, and clearer limitations.

“All of those have been edited accordingly based on all the detailed comments from above from the same reviewer 2. Please see the details above individually.”

I do not think extensive new field sampling is required for publication, provided the authors substantially revise the analysis and temper the claims. However, additional analyses using existing data are necessary: reduced versus full database concordance, culture/metagenomics comparison for V. cholerae, CTX sensitivity analysis, E. coli virulence/pathotype screening, AMR gene-level reporting, and clearer handling of controls/contamination. With these revisions, the manuscript would make a useful contribution to the field of neglected tropical disease diagnostics, outbreak genomics, and public health microbiology.

“We agree, we simply can not add more field samples. The field experiment requires long-term planning and is way too costly. For the remainder of these points, all of those have been edited accordingly based on all the detailed comments from above from the same reviewer 2. Please see the details above individually.”

Reviewer #3: General comments

The authors Jensen et al, carried out a study in which they evaluated a mobile, culture-independent metagenomic sequencing workflow for on-site detection of gastrointestinal pathogens directly from faecal samples collected from three health facilities in Burundi. The facilities were one (1) health centre, one (1) hospital and one (1) refugee transit camp. Furthermore, the authors state that they also collected one Stool samples and environmental samples were collected and analysed from samples from one (1) communal latrine, from one (1) open drain and one (1) river. The authors found out that V. cholerae signals were only detected in a subset of suspected cholera cases, while many samples were dominated by alternative bacterial taxa. The authors concluded that Mobile, offline metagenomic sequencing enables rapid frontline characterization of gastrointestinal disease, especially cholera-suspected. This is an interesting finding that has potential to attract readers and policy markers especially those in resource limited countries such as those in sub-Saharan Africa.

“We thank the reviewer for finding it interesting and with potential.”

However, there are major flaws with the study methods that affects its usability and replicability. Specific issues are below.

Major issues

1. Page 6, lines 145-161, Materials and Methods: Sample collection and study design. The authors include a sub-heading titled “Study design” yet they do not specify the epidemiological design that underpins this work. The type of design such as cross-sectional, cohort or case-control is not stated, and other essential elements required for replication are missing such as sample size and how it was derived, sampling frame, inclusion and exclusion criteria, sampling method. Without a clearly defined design, the study does not meet basic reporting standards such as STROBE Item 4 and ICMJE recommendations, which require explicit description of the design to allow other scientists to repeat the study under comparable conditions.

“We agree that a subsection entitled “Study design” should explicitly describe the study type and recruitment procedure, and that the original section did not do so sufficiently. The section has therefore been retitled “Study design, participant recruitment and sample collection.”

We now describe the study as a prospective, multi-site, descriptive field-feasibility and technology-evaluation study with single timepoint sampling during predefined field deployments (LINES 163-172). Participants were recruited using time-bounded, site-based availability sampling from eligible individuals present during each site visit and who were able to provide a faecal sample. Samples were not selected according to disease severity, anticipated pathogen status or subsequent sequencing results.

The revised Methods now define the clinical categories used at each site, the recruitment periods, eligibility and exclusion procedures, and the factors determining the achieved sample numbers (LINES 163-172). No formal epidemiological sample size or power calculation was performed because the study was designed to evaluate operational feasibility and analytical concordance rather than prevalence, diagnostic accuracy or population-level aetiology, which the text now is revised to reflect this. The sample numbers instead reflected the eligible participants and samples available during each field window, together with the operational capacity of the mobile workflow, including flowcell availability and field logistics.

We have also added a sample flow diagram (see new Figure S1) and a per-sample analytical summary showing who was sampled and which analyses were performed (Table S3). These revisions address the reporting principles underlying STROBE Item 4 and the ICMJE recommendations by explicitly stating the study type and providing sufficient detail on participant selection, sampling and analysis for the field evaluation to be understood and reproduced. We do not, however, present the study as a cohort, case-control or formal diagnostic-accuracy study (LINES 169-174).”

2. Page 5, lines 120–123: “In Burundi, cholera (Vibrio cholerae) is considered an endemic disease, but with occasional outbreaks… inadequate sanitation, limited access to clean drinking water, and natural disasters.” The authors correctly state cholera is endemic in Burundi hence suspected cases should be frequently seen. Page 6, lines 152–157: The authors report investigating only 31 suspected cholera cases at Rugombo Health Centre but do not state the target population, sampling frame, or selection method. Without this, it is unclear if the 31 cases represent complete enumeration or a non-random subset, which prevents assessment of selection bias. The authors should specify the sampling approach, case definition of a suspected cholera case, and rationale for 31 in Rugombo health centre or 9 in Rutana district being sufficient for their aims. Otherwise, the authors should limit conclusions to a descriptive report of these suspected cases only. The same explanation apply to the 21 cases investigated in Cishemere refugee transit centre district hospital.

“We agree that the sampling procedure, study population and clinical categories should have been described more explicitly. We have therefore added the recruitment periods, sampling approach, eligibility procedure and definitions used at each site to the revised Methods (All material and method section).

This was not a population-based epidemiological study designed to estimate cholera prevalence, etiological fractions or diagnostic performance. It was a prospective, multi-site field-feasibility and technology-evaluation study assessing whether an end-to-end, offline metagenomic workflow could be implemented directly at frontline sites under real-world conditions. The study population therefore comprised eligible individuals attending the selected sites during the predefined field-deployment periods, rather than the general population of Burundi.

At Rugombo, eligible participants were individuals classified by the attending healthcare professionals as patients with suspected cholera and managed through the Cholera Treatment Centre. At Rutana, participants were patients identified by clinical staff as presenting with gastrointestinal complaints. At Cishemere, the 21 participants comprised 12 individuals reporting stomach discomfort and nine individuals without reported gastrointestinal symptoms; they were not 21 patients with suspected gastrointestinal infection, all of this is now adjusted and included in the main text (Lines 214-218, 477-487).

The sites and deployment periods were planned in advance. Participants were enrolled from eligible individuals present during the time-limited sampling windows who provided a faecal sample. Samples were not selected according to disease severity, anticipated pathogen status or subsequent sequencing results. The 31 Rugombo patient samples, nine Rutana patient samples and 21 Cishemere samples therefore represent the samples available during these deployment periods and processable within the operational capacity of the mobile workflow. Thus the achieved sample numbers reflect the individuals and samples available during the deployment periods and the substantial practical constraints of conducting this work at remote field sites, including difficult road access, limited time at each location, sample availability, maintenance of the cold chain, flowcell capacity, and the need to perform DNA extraction, library preparation, sequencing and analysis on site, those are also now added in the main text (Lines 174-178).

No formal epidemiological sample-size or power calculation was performed for the reasons mentioned above. The achieved sample numbers were sufficient to demonstrate execution of the complete workflow across three distinct field settings, but not to support population-level inference. We now state this explicitly, acknowledge the potential for selection bias associated with time-bounded site-based recruitment, and limit the conclusions to the samples and settings investigated (Limitation section 648-663).”

3. Page 7, lines 145–168, Materials and Methods: The authors report samples from Rutana District 9, Cishemere Refugee Transit Camp 21, Rugombo Health Centre 36, and n=1 each from Rugombo open drainage, latrine, and river. The rationale for selecting only one site or facility per environmental source is not stated. Latrines, drainage points, and rivers occur as multiple units per community, and one health centre cannot represent district care-seeking. With n=1 per environmental source there is no variance, so results lack precision and external validity. Unequal allocation without a stated sampling frame introduces selection bias and precludes valid clinical vs environmental comparisons. The authors must define the target population, sampling frame, selection method, and justification for site-specific sample sizes. It is not clear why the authors preferred n=1 in terms of sites and environmental samples. Conclusions should therefore be limited to descriptive reporting of the specific units investigated.

“We completely agree with the reviewer that the environmental sampling was limited and cannot support estimates of variance, validity or formal comparisons between clinical and environmental samples. The three environmental samples were intended only as exploratory, descriptive samples from nearby potential exposure points in the Rugombo area, rather than as a representative environmental survey.

Broader environmental sampling was absolutely not feasible during the field deployment. A large crowd gathered around the team during sampling, creating operational and safety concerns. For this reason, sampling was restricted to the three environmental locations that could be reached safely on foot: one communal latrine, one open drain and one river-access point. Additional sites would have required travel over roads that were difficult to access and would have increased the safety risk to the field team.

We have now clarified the rationale and selection procedure in the Methods and explicitly state that these samples were selected on the basis of proximity, accessibility and safety (Lines 356-360). We have also further moderated the Results and Discussion so that the environmental findings are presented only as descriptive observations from the specific locations sampled. No clinical–environmental comparison or claim of representativeness is made, and we identify the absence of spatial and temporal replication as an important limitation (see limitation section Lines 648-663).”

4. Page 4, lines 101–104, Introduction: The authors state: “To address these diagnostic constraints, there is growing interest in approaches that can provide rapid, culture-independent identification of gastrointestinal infection agents, including V. cholerae, directly from clinical samples, while remaining deployable in resource-limited and outbreak-prone settings.” This frames the study as validating a rapid, culture-independent method for resource-limited settings. However, the manuscript does not present data supporting suitability for such settings. There is no analysis of reagent, equipment, or operational costs, and no comparison to standard-of-care or gold-standard methods on cost, infrastructure, or turnaround time. The results instead report performance metrics that reflect general analytical validity, not resource constraints. Thus, the claim of applicability to resource-limited settings is unsupported by the data presented.

“We agree that the original wording could be interpreted as claiming economic affordability or superiority to standard-of-care diagnostics, which was not evaluated in this study. We have therefore clarified the scope of the claim. The study directly demonstrates operational deployability under infrastructure limited field conditions: DNA extraction, library preparation, sequencing, basecalling and frontline analysis were performed on site without grid power, internet access or a centralised laboratory. The principal pathogen and AMR profiles were generally available within approximately 12 hours, although sequencing was continued for longer to increase depth and improve detection of lower-abundance signals.

The workflow is intended as a complementary escalation tool during outbreak investigations, particularly where rapid diagnostic tests are unavailable, negative or inconclusive, or where the causative pathogen is unknown. Unlike targeted rapid tests, the metagenomic approach can simultaneously detect V. cholerae, alternative gastrointestinal pathogens and AMR determinants without prior specification of a target. It also avoids delays associated with transporting samples to a central laboratory and waiting for culture-based identification.

We must also note that culturing should also not be regarded as an infallible gold standard in this context. Recovery depends on organism viability, prior antimicrobial exposure, transport and incubation conditions, and the use of appropriate selective media; fastidious, low-abundance or viable-but-non-culturable organisms may therefore be missed. By analysing total sample DNA directly, metagenomics can detect a broader range of organisms without requiring successful cultivation, although culture remains important for isolate recovery and phenotypic susceptibility testing. Additionally, rapid antigen tests are faster than either method but are targeted to *V. cholerae* O1/O139. Our data illustrate the consequence directly, as one of our isolates (R24) was non-O1/non-O139 and would not have been detected, and the diarrhoeagenic *E. coli* virulence markers we identified in 11 of 15 *E. coli*-dominated samples are outside the scope of any cholera-specific test. At outbreak onset, in unusual clusters, or where rapid tests are negative but illness persists, there is no target on which to base a targeted assay, and an untargeted approach is the only option available.

On cost, we have added an indicative consumables-cost benchmark to the Discussion. A recent study of a comparable ONT workflow estimated approximately €72 per sample at 12-plex and €39 per sample at 24-plex, with the flow cell accounting for most of the consumable cost (<https://www.frontiersin.org/journals/public-health/articles/10.3389/fpubh.2026.1755877/full> ). Because different extraction and library-preparation kits were used, these figures are presented only as contextual benchmarks (Lines 665-671).

We have not presented a formal cost-effectiveness comparison with culture or rapid tests, because personnel time, equipment amortisation, transport, shipping and local supply-chain costs were not measured and vary substantially between settings. The revised manuscript therefore uses “resource-limited settings” to refer specifically to independence from grid power, internet connectivity and centralised laboratory infrastructure, rather than to lower per-sample cost. We have also moderated the conclusions accordingly.”

Other comments

5. Page 16–17, lines 384–394, Figure 4 and legend and lines 397–409: The figures are clear and the data are valid, but they are irrelevant to the study aim. The goal is a rapid diagnostic test for gastrointestinal infections that works in resource-limited settings. Detecting V. cholerae and other pathogens is valid data. Also CTXφ confirmation shows the platform works in the lab, not that it is rapid or field-deployable. The authors do not compare to culture, the standard of care, and they report no sensitivity, specificity, positive or negative predictive value. There is also no data on cost per test. Hence, claims about resource-limited use should be removed until comparison to culture or Gold standard are provided.

“We still think that Figure 4 is relevant to the study aim. The primary aim was to evaluate whether the offline metagenomic workflow could be implemented at frontline sites, while secondary aims included broader pathogen detection and analytical comparison with comprehensive analysis. Figure 4 demonstrates application of the same workflow at two additional field settings, a district hospital and a refugee transit camp, and includes a direct comparison between the reduced laptop-based and comprehensive workflows at Rutana. It therefore supports both the multi-site field feasibility and analytical-concordance objectives. Nevertheless, we have simplified Figure 4 and moved less central exploratory analyses to the Supplementary Information to make its relevance clearer.

We agree that CTXφ detection should not be presented as evidence that the workflow itself is rapid or field-deployable. The CTXφ analysis was a secondary analysis used to assess the biological context and toxigenic potential of the detected V. cholerae signals. CTXφ was detected in 10 of the 11 samples with high-abundance V. cholerae, and the result was concordant with the main metagenomic findings. It is not used as evidence of turnaround time or field feasibility.

For V. cholerae, all 31 Rugombo patient samples underwent ABPW enrichment and TCBS culture, and we have now added the requested per-sample comparison and 2 × 2 agreement table (Table S7). We report positive, negative and overall percent agreement rather than sensitivity, specificity, positive predictive value or negative predictive value because the study was not designed or powered as a formal diagnostic-accuracy evaluation. In addition, TCBS culture is an imperfect comparator rather than an infallible gold standard. It detects only organisms that grow under the applied conditions, and no single culture method can serve as a universal reference standard for the broader range of gastrointestinal pathogens detectable by an untargeted metagenomic workflow. The revised manuscript therefore distinguishes presumptive TCBS growth, sequencing-supported culture recovery and recovery of a pure isolate (lines 443-460).

Regarding field deployment, the study directly demonstrates that DNA extraction, library preparation, sequencing, basecalling and frontline analysis could be performed on site without grid power, internet access or centralised laboratory infrastructure. Initial pathogen and AMR profiles were generally obtained within approximately 12 hours; longer sequencing runs were continued to increase depth rather than because the initial result required the complete run.

We have also added an indicative consumables-cost benchmark from a comparable ONT workflow, while explicitly stating that no formal cost-effectiveness comparison with culture or rapid tests was performed. We have accordingly narrowed rather than removed the resource-limited-setting claim: it refers to demonstrated independence from grid power, internet access and centralised laboratory infrastructure, not to proven lower unit cost or superiority to standard-of-care diagnostics (Lines 673-684).”

6. Across the manuscript: The authors use “suspected cholera” and “gastrointestinal symptoms” throughout but never define them. Undefined case definitions introduce speculation and limit reproducibility. To avoid ambiguity the authors should specify diagnostic criteria for both terms.

“We agree that these clinical categories required clearer definition. In the revised Methods, “patients with suspected cholera” refers to individuals classified as suspected cholera cases by the attending healthcare professionals and managed through the Cholera Treatment Centre before sequencing results were available. “Patients with gastrointestinal symptoms” refers to individuals identified by the attending clinical staff as presenting with a gastrointestinal complaint or illness; this was a broader clinical category and was not intended to represent a single standardised syndrome.

Detailed symptom information, including stool appearance, dehydration status, symptom duration, treatment and prior antibiotic exposure, was not recorded systematically across all sites. We have stated this explicitly rather than applying diagnostic criteria that were not used during recruitment, and we identify the limited clinical metadata as a study limitation (Lines 219-230).”

END.

**Potential Copyright Issues:**

i) Figure Fig 1. Please confirm whether you drew the images / clip-art within the figure panels by hand. If you did not draw the images, please provide (a) a link to the source of the images or icons and their license / terms of use; or (b) written permission from the copyright holder to publish the images or icons under our CC BY 4.0 license. Alternatively, you may replace the images with open source alternatives. See these open source resources you may use to replace images / clip-art: - https://commons.wikimedia.org - https://openclipart.org/.

“All icons, illustrations and schematic components in Figure 1 were created by the first author specifically for this manuscript. No third-party clip art or copyrighted images were used.”

ii) Figure Fig 2. Please (a) provide a direct link to the base layer of the map (i.e., the country or region border shape) and ensure this is also included in the figure legend; and (b) provide a link to the terms of use / license information for the base layer image or shapefile. We cannot publish proprietary or copyrighted maps (e.g. Google Maps, Mapquest) and the terms of use for your map base layer must be compatible with our CC BY 4.0 license. Note: if you created the map in a software program like R or ArcGIS, please locate and indicate the source of the basemap shapefile onto which data has been plotted. If your map was obtained from a copyrighted source please amend the figure so that the base map used is from an openly available source. Alternatively, please provide explicit written permission from the copyright holder granting you the right to publish the material under our CC BY 4.0 license. If you are unsure whether you can use a map or not, please do reach out and we will be able to help you. The following websites are good examples of where you can source open access or public domain maps: * U.S. Geological Survey (USGS) - All maps are in the public domain. (http://www.usgs.gov) * PlaniGlobe - All maps are published under a Creative Commons license so please cite u201cPlaniGlobe, http://www.planiglobe.com, CC BY 2.0u201d in the image credit after the caption. (http://www.planiglobe.com/?lang=enl) * Natural Earth - All maps are public domain. (http://www.naturalearthdata.com/about/terms-of-use/).

“The map was created with the QGIS software, which we now reference in the method section. The map utilises a basemap from ©OpenStreetMap contributors available under the Open Database License, and country boundary data from Natural Earth, which is also public domain. We have explained this in the method. ”

--------------------------------------------------

We look forward to hearing from you in due course.

Kind regards,

Divya Selvaraj

Editorial Office

On behalf of PLOS Neglected Tropical Diseases
