# Supplementary material for: Syndromic cholera diagnosis masks diverse causes of diarrhoeal disease in Burundi revealed by portable metagenomics
Source: PLoS Negl Trop Dis. 2026 Sep 1;20(9):e0014175. doi: 10.1371/journal.pntd.0014175 (PMC13557499; doi:10.1371/journal.pntd.0014175)
Supplement: Peer review history

# Peer review: Decision Letter 0

**DOI:** 10.1371/journal.pntd.0014175.r001

**Authors:** Jeffrey H Withey, Ana LTO Nascimento

6 Jul 2026

PNTD-D-26-00557

Syndromic cholera diagnosis masks diverse causes of diarrhoeal disease in Burundi revealed by portable metagenomics

PLOS Neglected Tropical Diseases

Dear Dr. Otani,

Thank you for submitting your manuscript to PLOS Neglected Tropical Diseases. After careful consideration, we feel that it has merit but does not fully meet PLOS Neglected Tropical Diseases's publication criteria as it currently stands. Therefore, we invite you to submit a revised version of the manuscript that addresses the points raised during the review process.

Please submit your revised manuscript within in three month. If you will need more time than this to complete your revisions, please reply to this message or contact the journal office at plosntds@plos.org. When you're ready to submit your revision, log on to https://www.editorialmanager.com/pntd/ and select the 'Submissions Needing Revision' folder to locate your manuscript file.

Please include the following items when submitting your revised manuscript:

* A letter that responds to each point raised by the editor and reviewer(s). You should upload this letter as a separate file labeled 'Response to Reviewers'. This file does not need to include responses to any formatting updates and technical items listed in the 'Journal Requirements' section below.

* A marked-up copy of your manuscript that highlights changes made to the original version. You should upload this as a separate file labeled 'Revised Manuscript with Track Changes'.

* An unmarked version of your revised paper without tracked changes. You should upload this as a separate file labeled 'Manuscript'.

If you would like to make changes to your financial disclosure, competing interests statement, or data availability statement, please make these updates within the submission form at the time of resubmission. Guidelines for resubmitting your figure files are available below the reviewer comments at the end of this letter.

As the corresponding author, your ORCID iD is verified in the submission system and will appear in the published article. PLOS supports the use of ORCID, and we encourage all coauthors to register for an ORCID iD and use it as well. Please encourage your coauthors to verify their ORCID iD within the submission system before final acceptance, as unverified ORCID iDs will not appear in the published article. Only the individual author can complete the verification step; PLOS staff cannot verify ORCID iDs on behalf of authors.

We look forward to receiving your revised manuscript.

Kind regards,

Jeffrey H Withey

Academic Editor

PLOS Neglected Tropical Diseases

Ana LTO Nascimento

Section Editor

PLOS Neglected Tropical Diseases

Shaden Kamhawi

co-Editor-in-Chief

PLOS Neglected Tropical Diseases

orcid.org/0000-0003-4304-636XX

Paul Brindley

co-Editor-in-Chief

PLOS Neglected Tropical Diseases

orcid.org/0000-0003-1765-0002

**Additional Editor Comments (if provided):**

The reviewers generally agreed that this is an important study and that there were positives in the manuscript. However, there were issues raised by two of the reviewers that should be addressed in the revised manuscript. Please respond point by point to their comments and submit this with your revised manuscript.

**Journal Requirements:**

If the reviewer comments include a recommendation to cite specific previously published works, please review and evaluate these publications to determine whether they are relevant and should be cited. There is no requirement to cite these works unless the editor has indicated otherwise.

1) Please ensure that the CRediT author contributions listed for every co-author are completed accurately and in full.

At this stage, the following Authors/Authors require contributions: Emilie Egholm Bruun Jensen, Néhémie NZOYIKORERA, Mirena Ivanova, Pimlapas Leekitcharoenphon, Marie Noelle UWINEZA, Idrissa Diawara, Joseph Nyandwi, Frank M. Aarestrup, and Saria Otani. Please ensure that the full contributions of each author are acknowledged in the "Add/Edit/Remove Authors" section of our submission form.

The list of CRediT author contributions may be found here: https://journals.plos.org/plosntds/s/authorship#loc-author-contributions

2) Please upload all main figures as separate Figure files in .tif or .eps format. For more information about how to convert and format your figure files please see our guidelines:

https://journals.plos.org/plosntds/s/figures

3) We have noticed that you have uploaded Supporting Information files, but you have not included a list of legends. Please add a full list of legends for your Supporting Information files after the references list.

4) Please amend your detailed Financial Disclosure statement. This is published with the article. It must therefore be completed in full sentences and contain the exact wording you wish to be published.

1) State the initials, alongside each funding source, of each author to receive each grant. For example: "This work was supported by the National Institutes of Health (####### to AM; ###### to CJ) and the National Science Foundation (###### to AM)."

2) State what role the funders took in the study. If the funders had no role in your study, please state: "The funders had no role in study design, data collection and analysis, decision to publish, or preparation of the manuscript."

3) If any authors received a salary from any of your funders, please state which authors and which funders..

If you did not receive any funding for this study, please simply state: u201cThe authors received no specific funding for this work.u201d

5) We have amended your Competing Interest statement to comply with journal style. We kindly ask that you double check the statement and let us know if anything is incorrect.

6) Thank you for stating "The sequencing data generated during the study is available through the European Nucleotide Archive (ENA) repository: PRJEB108124. This includes metagenomic sequencing reads as well as reads and assemblies of five V. cholerae single isolates, including the circularised non O1/O139 strain (R24). " However, we were unable to access the sequence data using the information provided. Please review and update the Data Availability Statement to ensure that the underlying data are publicly accessible and that all accession numbers, links, or repository details are correct and functional.

**Reviewers' Comments:**

Reviewer's Responses to Questions

**Key Review Criteria Required for Acceptance?**

As you describe the new analyses required for acceptance, please consider the following:

**Methods**

-Are the objectives of the study clearly articulated with a clear testable hypothesis stated?

-Is the study design appropriate to address the stated objectives?

-Is the population clearly described and appropriate for the hypothesis being tested?

-Is the sample size sufficient to ensure adequate power to address the hypothesis being tested?

-Were correct statistical analysis used to support conclusions?

-Are there concerns about ethical or regulatory requirements being met?

Reviewer #1: --In general, very clear and detailed.

--There are many places here where results are presented (line 152, 169-171, etc.). The results of how many cholera samples were taken belongs at the start of the results.

--May be helpful to add as an appendix all of the bacterial genera that could be detected?

Reviewer #2: The manuscript addresses a crucial and timely public health question: whether portable, offline metagenomic sequencing can enhance etiologic resolution among patients with clinically suspected cholera in resource-limited settings. The rationale is strong because the authors clearly describe the limitations of syndromic cholera diagnosis and centralized laboratory confirmation in Burundi and similar settings. The study objective is broadly stated as evaluating a mobile, culture-independent metagenomic workflow for on-site detection of Vibrio cholerae and other gastrointestinal pathogens directly from fecal samples. However, the manuscript would be stronger if the authors explicitly stated a primary testable hypothesis and separated the primary objective from secondary objectives. For example, the primary objective could be framed as evaluating the feasibility and concordance of an offline ONT-based workflow against culture and comprehensive bioinformatic analysis, while secondary objectives could include the detection of alternative bacterial taxa, CTX, AMR genes, and environmental signals. The current objective is clear in general terms, but it is not yet framed as a formal hypothesis-driven or diagnostic evaluation study.

The study design is appropriate as a field feasibility and proof-of-concept study, but it is not sufficiently powered or structured to support strong diagnostic accuracy, etiologic attribution, or public health implementation claims. The authors sampled 31 suspected cholera cases and five asymptomatic companions at Rugombo Health Centre, nine gastrointestinal-symptom patients at Rutana District Hospital, 12 individuals with stomach discomfort and nine asymptomatic individuals at Cishemere refugee camp, plus three environmental samples. This is a valuable field dataset, but the sampling appears opportunistic rather than consecutive or prospectively powered. The authors should clarify inclusion and exclusion criteria, whether all eligible patients during the sampling period were enrolled, how “suspected cholera,” “gastrointestinal symptoms,” and “stomach discomfort” were defined, and whether rapid diagnostic test, microscopy, culture, serology, PCR, dehydration status, stool appearance, antibiotic exposure, age, sex, symptom duration, and treatment information were available.

The sample size is sufficient for a pilot feasibility demonstration but not sufficient for estimating diagnostic performance, pathogen prevalence, or etiologic fractions with precision. No sample size justification or power calculation is provided. This is acceptable if the study is explicitly presented as a field feasibility study, but the authors should avoid language implying definitive diagnostic accuracy or generalizable epidemiologic conclusions.

The laboratory and bioinformatic methods are promising and mostly described in useful detail, including DNA extraction, ONT library preparation, flow cells, basecalling, demultiplexing, host-read removal, reduced-database KMA mapping, AMR screening, culture confirmation, isolate sequencing, and comprehensive HPC-based analyses. However, several methodological clarifications are required for acceptance. First, the authors should provide explicit pathogen-calling thresholds for the frontline workflow and justify the very permissive KMA identity threshold of 25% and minimum depth of 1. Second, they should present concordance between the reduced laptop database and the full HPC database, ideally as a per-sample comparison table with agreement, discordance, and reasons for discordance. Third, if culture was used as confirmation for Rugombo samples, a simple 2×2 table comparing metagenomics and culture for V. cholerae should be provided, with exact binomial confidence intervals where appropriate. If culture, Sero grouping, or PCR were not performed for all samples, this limitation should be explicitly stated.

Statistical analysis requires strengthening. The use of alpha diversity, CLR transformation, PCA, ALDEx2, Welch’s tests with FDR correction, and Pearson correlations is generally reasonable for exploratory microbiome analysis; however, the manuscript would benefit from a clearer distinction between descriptive, exploratory, and confirmatory analyses. The authors should report effect estimates with uncertainty intervals wherever possible. The CTXϕ correlation should include the number of samples included, p-value, confidence interval, sensitivity to zeros/outliers, and ideally a Spearman or permutation-based sensitivity analysis. Differential abundance testing among very small groups should be presented cautiously and should include sample sizes per comparison.

A major concern is the interpretation of E. coli. The manuscript frequently refers to E. coli as a potential pathogen based on its abundance. However, E. coli is also a common gut commensal, and metagenomic species-level detection alone is insufficient to infer diarrheagenic E. coli. The authors should screen for and report diarrheagenic E. coli virulence markers or pathotype-associated genes, such as stx1/stx2, eae, bfpA, elt, est, ipaH, aggR, and related markers. Without these analyses, the manuscript should avoid implying that E. coli abundance alone indicates causation.

AMR analyses also require more detail. The authors should report gene-level AMR findings, identity and coverage thresholds, whether genes were assembled or read-based only, and whether any AMR genes could be linked to specific taxa or contigs. Stool metagenomic AMR detection provides useful surveillance information but should not be interpreted as direct clinical susceptibility evidence unless validated.

The ethics statement reports approval by the National Ethics Committee in Burundi and a waiver of consent, with samples collected by health professionals. This is acceptable in principle, but because the study includes symptomatic patients, asymptomatic companions, and a refugee transit camp population, the authors should provide more detail on the waiver rationale, de-identification, handling of vulnerable populations, and whether environmental sampling required separate permissions.

Reviewer #3: -Are the objectives of the study clearly articulated with a clear testable hypothesis stated? YES

-Is the study design appropriate to address the stated objectives? NO. STUDY DESIGN NOT GIVEN

-Is the population clearly described and appropriate for the hypothesis being tested? NO.

-Is the sample size sufficient to ensure adequate power to address the hypothesis being tested? NO. SEE THE DETAILED REPORT.

-Were correct statistical analysis used to support conclusions? NO.

-Are there concerns about ethical or regulatory requirements being met? NO

**********

**Results**

-Does the analysis presented match the analysis plan?

-Are the results clearly and completely presented?

-Are the figures (Tables, Images) of sufficient quality for clarity?

Reviewer #1: --Curious why Bacteroides was determined to be non-pathogenic?

--Can the authors distinguish which of the bacteria the AMR genes were linked to? Some of these are likely plasmid mediated and not necessarily able to be linked.

Reviewer #2: The results generally follow the stated field-evaluation approach, but because the manuscript does not clearly define a formal analysis plan, it is difficult to determine which results were primary versus exploratory. The sequencing output section is useful and transparent, but the authors should add a consolidated table showing per-sample read counts, human-read percentage, bacterial-read count, dominant taxon, pathogen call, culture result, CTX result, AMR summary, and whether the reduced and full databases agreed.

The main finding that V. cholerae was detected in 11 of 31 suspected cholera cases, while other samples were dominated by other taxa, especially E. coli, is important and relevant. However, the result should be presented with more diagnostic structure. The manuscript should clearly show how many suspected cholera cases were positive by frontline metagenomics, comprehensive metagenomics, culture, serogroup/serotype testing, and CTX detection. The current presentation is promising but requires clearer quantitative alignment between platforms and methods.

The figures are informative but need improvement for clarity. Figure 1 helps show the workflow and field sites. Figure 2 provides useful, sequencing quality-control information. Figure 3 is central to the manuscript, but it is visually dense, and the sample labels, pathogen-call indicators, and relative-abundance bars are difficult to interpret quickly. Figure 4 is also useful but should be clarified with larger labels, clearer color palettes, and more explicit separation of symptomatic, asymptomatic, and hospital samples. The authors should consider adding a simpler diagnostic summary figure or heatmap that directly compares culture, frontline metagenomics, comprehensive metagenomics, CTX, and AMR findings.

The CTX result is one of the strongest biological supports for the V. cholerae interpretation, but it is currently underdeveloped. The authors should present the CTX correlation as a main figure or expanded supplementary figure with sample labels, correlation statistics, p-values, confidence intervals, and sensitivity analyses. This would make the claim that CTX helps distinguish toxigenic signal from background detection much more convincing.

The AMR results are potentially valuable but currently too general. Reporting resistance classes alone is insufficient. The authors should provide gene-level AMR calls and avoid overinterpreting clinical implications unless phenotype or organism-specific linkage is available.

Reviewer #3: -Does the analysis presented match the analysis plan? NO

-Are the results clearly and completely presented? YES

-Are the figures (Tables, Images) of sufficient quality for clarity? YES

**********

**Conclusions**

-Are the conclusions supported by the data presented?

-Are the limitations of analysis clearly described?

-Do the authors discuss how these data can be helpful to advance our understanding of the topic under study?

-Is public health relevance addressed?

Reviewer #1: --Very nice statement in lines 437-429.

--Very strong conclusions supported by the results.

Reviewer #2: The core conclusion that portable offline metagenomics is feasible in field conditions and can provide broader etiologic insight than syndromic cholera diagnosis is supported by the data. The study is novel, operationally relevant, and well aligned with the public health needs of cholera-prone and resource-limited settings.

However, several conclusions should be moderated. The data support feasibility and descriptive detection, but not definitive diagnostic accuracy, pathogen causality, or clinical management impact. Claims about “alternative etiologies” should be softened unless virulence/pathotype evidence is added for organisms such as E. coli. Detection of a bacterial species in stool does not necessarily establish disease causation.

The authors do acknowledge important limitations, including minimal clinical metadata, lack of a prospective detailed comparison against serotype testing and culture for all samples, and limited environmental sampling. These limitations are appropriate but should be expanded and moved into a more explicit limitations paragraph. Additional limitations should include small sample size, non-random/opportunistic sampling, lack of power calculation, limited controls, possible contamination or barcode crosstalk in multiplexed metagenomics, limited ability to detect RNA viruses if only DNA sequencing was performed, and inability to attribute AMR genes to specific pathogens without assembly/linkage evidence.

The public health relevance is strong. The manuscript directly addresses outbreak response, delayed centralized diagnostics, and diagnostic uncertainty in suspected cholera cases. The authors should more clearly explain how this workflow fits into existing cholera response systems: for example, as an escalation tool when rapid tests are negative/inconclusive, during early outbreak confirmation, for sentinel surveillance, or for investigation of unusual clusters.

Reviewer #3: -Are the conclusions supported by the data presented? NO. SEE THE REPORT

-Are the limitations of analysis clearly described? NO. NOTHING ON THE COST YET THE STUDY IS RESOURCE LIMITED SETTING.

-Do the authors discuss how these data can be helpful to advance our understanding of the topic under study? YES

-Is public health relevance addressed? YES

**********

**Editorial and Data Presentation Modifications?**

Use this section for editorial suggestions as well as relatively minor modifications of existing data that would enhance clarity. If the only modifications needed are minor and/or editorial, you may wish to recommend “Minor Revision” or “Accept”.

Reviewer #1: --Please be sure that genus/species names are italicized throughout.

--I would suggest avoiding the term "cholera-suspected diarrhea"; you could use acute watery diarrhea or another term that is more widely accepted and does not include the genus name.

--Patients are not "suspected of cholera" but instead they are patients "with suspected cholera."

--Line 93, is missing an "and" before time

--Line 120, should be "cholera" not cholerae when talking about the disease. The species is cholerae.

--Line 292 and line 299 are missing a period at the end of the sentence after the parentheses.

Reviewer #2: Several editorial revisions are needed before acceptance.

First, the manuscript needs careful copyediting. Examples include “reply heavily” instead of “rely heavily,” inconsistent use of “cholerae” versus “cholera,” inconsistent spelling of “faecal/fecal” and “diarrhoeal/diarrheal,” “lap-top” versus “laptop,” and awkward phrases such as “sufficient high amount of reads” and “the absence of detections could be difficult.” The authors should also avoid referring to “cholerae-negative patients”; “V. cholerae-negative patients” would be clearer.

Second, the authors should define all major abbreviations at first use, including CTX, ONT, KMA, AMR, CLR, HPC, TCBS, ABPW, ST, and FDR. The manuscript should also consistently italicize bacterial species names.

Third, the title is strong but slightly broad. Because the study is a pilot field evaluation rather than a definitive etiologic study, the authors may consider a more measured title such as: “Portable metagenomics reveals heterogeneous microbial profiles among suspected cholera cases in Burundi.”

Fourth, the data presentation should be improved with the following additions: a STARD-like flow diagram of sample inclusion and testing, a per-sample diagnostic summary table, a table comparing reduced-database and full-database classifications, a table of AMR genes with identity/coverage thresholds, and a runtime/cost/power table for field deployment. The authors state that the workflow can generate actionable profiles in approximately 12 hours, but the sequencing durations reported range from about 11 to 45 hours; this discrepancy should be reconciled clearly.

Fifth, the data availability statement is helpful and provides ENA accession. However, the authors should also provide scripts, pipeline parameters, reduced database construction details, database versioning, and ideally a reproducible workflow repository.

Reviewer #3: (No Response)

**********

**Summary and General Comments**

Use this section to provide overall comments, discuss strengths/weaknesses of the study, novelty, significance, general execution and scholarship. You may also include additional comments for the author, including concerns about dual publication, research ethics, or publication ethics. If requesting major revision, please articulate the new experiments that are needed.

Reviewer #1: --I am unsure what the authors intend by this statement: "...can be infected by a wide range of other pathogens that are not detected by standard diagnostics." Do the authors mean standard CHOLERA diagnostics? Because other pathogens certainly can be detected or suspected based on simple diagnostic tests on stool including stool wbc, stool wbc, or O&P.

--Would also add to this sentence that another concern (besides dehydration) is easy transmission in places with substandard hygiene. "Because severe dehydration can develop within hours with diarrhoeal diseases, outbreak control and patient management depend on timely recognition of true cholera cases and differentiating them from other causes of gastrointestinal illness with overlapping symptoms."

--Would be good in the introduction to acknowledge co-infection of cholera + another bacterial pathogen also.

--Very nice description in the introduction about the tests and Burundi, but please add a statement about aim/objective and the 3 populations that the test was deployed in.

Reviewer #2: This is a timely, innovative, and potentially important manuscript that demonstrates the feasibility of deploying portable ONT metagenomics for diarrheal disease investigation in Burundi. The study’s major strengths include its field-based implementation, relevance to cholera outbreak response, inclusion of multiple clinical settings, integration of frontline laptop-based analysis with deeper HPC confirmation, and culture/WGS follow-up for selected V. cholerae isolates. The manuscript is especially valuable because it addresses a real operational problem: syndromic cholera diagnosis can be nonspecific, and delayed centralized testing can limit timely public health action.

The main weaknesses are not conceptual but analytical and interpretive. The manuscript currently overstates some findings relative to the study design. It should be framed as a pilot feasibility and exploratory metagenomic field study rather than a fully validated diagnostic accuracy study. Required revisions include clearer objectives and hypotheses, stronger description of patient selection and clinical metadata, explicit pathogen-calling thresholds, diagnostic concordance tables, uncertainty estimates, improved CTX analysis, organism/pathotype-level interpretation of E. coli, gene-level AMR reporting, and clearer limitations.

I do not think extensive new field sampling is required for publication, provided the authors substantially revise the analysis and temper the claims. However, additional analyses using existing data are necessary: reduced versus full database concordance, culture/metagenomics comparison for V. cholerae, CTX sensitivity analysis, E. coli virulence/pathotype screening, AMR gene-level reporting, and clearer handling of controls/contamination. With these revisions, the manuscript would make a useful contribution to the field of neglected tropical disease diagnostics, outbreak genomics, and public health microbiology.

Reviewer #3: General comments

The authors Jensen et al, carried out a study in which they evaluated a mobile, culture-independent metagenomic sequencing workflow for on-site detection of gastrointestinal pathogens directly from faecal samples collected from three health facilities in Burundi. The facilities were one (1) health centre, one (1) hospital and one (1) refugee transit camp. Furthermore, the authors state that they also collected one Stool samples and environmental samples were collected and analysed from samples from one (1) communal latrine, from one (1) open drain and one (1) river. The authors found out that V. cholerae signals were only detected in a subset of suspected cholera cases, while many samples were dominated by alternative bacterial taxa. The authors concluded that Mobile, offline metagenomic sequencing enables rapid frontline characterization of gastrointestinal disease, especially cholera-suspected. This is an interesting finding that has potential to attract readers and policy markers especially those in resource limited countries such as those in sub-Saharan Africa. However, there are major flaws with the study methods that affects its usability and replicability. Specific issues are below.

Major issues

1. Page 6, lines 145-161, Materials and Methods: Sample collection and study design. The authors include a sub-heading titled “Study design” yet they do not specify the epidemiological design that underpins this work. The type of design such as cross-sectional, cohort or case-control is not stated, and other essential elements required for replication are missing such as sample size and how it was derived, sampling frame, inclusion and exclusion criteria, sampling method. Without a clearly defined design, the study does not meet basic reporting standards such as STROBE Item 4 and ICMJE recommendations, which require explicit description of the design to allow other scientists to repeat the study under comparable conditions.

2. Page 5, lines 120–123: “In Burundi, cholera (Vibrio cholerae) is considered an endemic disease, but with occasional outbreaks… inadequate sanitation, limited access to clean drinking water, and natural disasters.” The authors correctly state cholera is endemic in Burundi hence suspected cases should be frequently seen. Page 6, lines 152–157: The authors report investigating only 31 suspected cholera cases at Rugombo Health Centre but do not state the target population, sampling frame, or selection method. Without this, it is unclear if the 31 cases represent complete enumeration or a non-random subset, which prevents assessment of selection bias. The authors should specify the sampling approach, case definition of a suspected cholera case, and rationale for 31 in Rugombo health centre or 9 in Rutana district being sufficient for their aims. Otherwise, the authors should limit conclusions to a descriptive report of these suspected cases only. The same explanation apply to the 21 cases investigated in Cishemere refugee transit centre district hospital.

3. Page 7, lines 145–168, Materials and Methods: The authors report samples from Rutana District 9, Cishemere Refugee Transit Camp 21, Rugombo Health Centre 36, and n=1 each from Rugombo open drainage, latrine, and river. The rationale for selecting only one site or facility per environmental source is not stated. Latrines, drainage points, and rivers occur as multiple units per community, and one health centre cannot represent district care-seeking. With n=1 per environmental source there is no variance, so results lack precision and external validity. Unequal allocation without a stated sampling frame introduces selection bias and precludes valid clinical vs environmental comparisons. The authors must define the target population, sampling frame, selection method, and justification for site-specific sample sizes. It is not clear why the authors preferred n=1 in terms of sites and environmental samples. Conclusions should therefore be limited to descriptive reporting of the specific units investigated.

4. Page 4, lines 101–104, Introduction: The authors state: “To address these diagnostic constraints, there is growing interest in approaches that can provide rapid, culture-independent identification of gastrointestinal infection agents, including V. cholerae, directly from clinical samples, while remaining deployable in resource-limited and outbreak-prone settings.” This frames the study as validating a rapid, culture-independent method for resource-limited settings. However, the manuscript does not present data supporting suitability for such settings. There is no analysis of reagent, equipment, or operational costs, and no comparison to standard-of-care or gold-standard methods on cost, infrastructure, or turnaround time. The results instead report performance metrics that reflect general analytical validity, not resource constraints. Thus, the claim of applicability to resource-limited settings is unsupported by the data presented.

Other comments

5. Page 16–17, lines 384–394, Figure 4 and legend and lines 397–409: The figures are clear and the data are valid, but they are irrelevant to the study aim. The goal is a rapid diagnostic test for gastrointestinal infections that works in resource-limited settings. Detecting V. cholerae and other pathogens is valid data. Also CTXϕ confirmation shows the platform works in the lab, not that it is rapid or field-deployable. The authors do not compare to culture, the standard of care, and they report no sensitivity, specificity, positive or negative predictive value. There is also no data on cost per test. Hence, claims about resource-limited use should be removed until comparison to culture or Gold standard are provided.

6. Across the manuscript: The authors use “suspected cholera” and “gastrointestinal symptoms” throughout but never define them. Undefined case definitions introduce speculation and limit reproducibility. To avoid ambiguity the authors should specify diagnostic criteria for both terms.

END

**********

PLOS authors have the option to publish the peer review history of their article (what does this mean?). If published, this will include your full peer review and any attached files.

If you choose “no”, your identity will remain anonymous but your review may still be made public.

**Do you want your identity to be public for this peer review?**  For information about this choice, including consent withdrawal, please see our Privacy Policy.

Reviewer #1: No

Reviewer #2: No

Reviewer #3: No

[NOTE: If reviewer comments were submitted as an attachment file, they will be attached to this email and accessible via the submission site. Please log into your account, locate the manuscript record, and check for the action link "View Attachments". If this link does not appear, there are no attachment files.]

**Figure resubmission:**

**Reproducibility:**

To enhance the reproducibility of your results, we recommend that authors of applicable studies deposit laboratory protocols in protocols.io, where a protocol can be assigned its own identifier (DOI) such that it can be cited independently in the future. Additionally, PLOS ONE offers an option to publish peer-reviewed clinical study protocols. Read more information on sharing protocols at https://plos.org/protocols?utm_medium=editorial-email&utm_source=authorletters&utm_campaign=protocols

Attachment Submitted filename: Reviwer report on authors Jensen et al_ Reviewer Godfrey.pdf

# Author comment: Author response to Decision Letter 1

**DOI:** 10.1371/journal.pntd.0014175.r002

18 Aug 2026

Attachment Submitted filename: Jensen_Nzoyikorera_et_al_Response_Letter.docx

# Editor report: Decision Letter 1

**DOI:** 10.1371/journal.pntd.0014175.r003

**Authors:** Jeffrey H Withey, Ana LTO Nascimento

23 Aug 2026

Dear Dr. Otani,

We are pleased to inform you that your manuscript 'Syndromic cholera diagnosis masks diverse causes of diarrhoeal disease in Burundi revealed by portable metagenomics ' has been provisionally accepted for publication in PLOS Neglected Tropical Diseases. Thank you for your comprehensive and thoughtful responses to the reviewers' concerns.

Before your manuscript can be formally accepted you will need to complete some formatting changes, which you will receive in a follow up email. A member of our team will be in touch with a set of requests.

Please note that your manuscript will not be scheduled for publication until you have made the required changes, so a swift response is appreciated.

IMPORTANT: The editorial review process is now complete. PLOS will only permit corrections to spelling, formatting or significant scientific errors from this point onwards. Requests for major changes, or any which affect the scientific understanding of your work, will cause delays to the publication date of your manuscript.

Should you, your institution's press office or the journal office choose to press release your paper, you will automatically be opted out of early publication. We ask that you notify us now if you or your institution is planning to press release the article. All press must be co-ordinated with PLOS.

Thank you again for supporting Open Access publishing; we are looking forward to publishing your work in PLOS Neglected Tropical Diseases.

Best regards,

Jeffrey H Withey

Academic Editor

PLOS Neglected Tropical Diseases

Ana LTO Nascimento

Section Editor

PLOS Neglected Tropical Diseases

Shaden Kamhawi

co-Editor-in-Chief

PLOS Neglected Tropical Diseases

orcid.org/0000-0003-4304-636XX

Paul Brindley

co-Editor-in-Chief

PLOS Neglected Tropical Diseases

orcid.org/0000-0003-1765-0002

***********************************************************

p.p1 {margin: 0.0px 0.0px 0.0px 0.0px; line-height: 16.0px; font: 14.0px Arial; color: #323333; -webkit-text-stroke: #323333}span.s1 {font-kerning: none

# Editor report: Acceptance letter

**DOI:** 10.1371/journal.pntd.0014175.r004

**Authors:** Jeffrey H Withey, Ana LTO Nascimento

Dear Dr Otani,

We are delighted to inform you that your manuscript, "Syndromic cholera diagnosis masks diverse causes of diarrhoeal disease in Burundi revealed by portable metagenomics ," has been formally accepted for publication in PLOS Neglected Tropical Diseases.

We have now passed your article onto the PLOS Production Department who will complete the rest of the publication process. All authors will receive a confirmation email upon publication.

The corresponding author will soon be receiving a typeset proof for review, to ensure errors have not been introduced during production. Please review the PDF proof of your manuscript carefully, as this is the last chance to correct any scientific or type-setting errors. Please note that major changes, or those which affect the scientific understanding of the work, will likely cause delays to the publication date of your manuscript. Note: Proofs for Front Matter articles (Editorial, Viewpoint, Symposium, Review, etc...) are generated on a different schedule and may not be made available as quickly.

Soon after your final files are uploaded, the early version of your manuscript will be published online unless you opted out of this process. The date of the early version will be your article's publication date. The final article will be published to the same URL, and all versions of the paper will be accessible to readers.

For Research Articles, you will receive an invoice from PLOS for your publication fee after your manuscript has reached the completed accept phase. If you receive an email requesting payment before acceptance or for any other service, this may be a phishing scheme. Learn how to identify phishing emails and protect your accounts at https://explore.plos.org/phishing.

Thank you again for supporting open-access publishing; we are looking forward to publishing your work in PLOS Neglected Tropical Diseases.

Best regards,

Shaden Kamhawi

co-Editor-in-Chief

PLOS Neglected Tropical Diseases

Paul Brindley

co-Editor-in-Chief

PLOS Neglected Tropical Diseases
